# M4205 (IDRX-42) Is a Highly Selective and Potent Inhibitor of Relevant Oncogenic Driver and Resistance Variants of KIT in Cancer

**DOI:** 10.1158/1535-7163.MCT-24-0699

**Published:** 2025-02-28

**Authors:** Christina Esdar, Nina Linde, Andreas Blum, Hanno Schieferstein, Christine Drechsler, Eva Sherbetjian, Carl Petersson, Edith Ross, Birgitta Leuthner, Ulrich Grädler, Dieter Dorsch, Andree Blaukat

**Affiliations:** The healthcare business of Merck KGaA, Darmstadt, Germany.

## Abstract

Primary activating mutations in *KIT* (exon 9/11) are key driver alterations in about 80% of gastrointestinal stromal tumors (GIST). Imatinib, a small-molecule tyrosine kinase inhibitor, is used successfully as first-line therapy for patients with unresectable metastatic or recurrent GIST, but secondary resistance mutations in the *KIT* kinase domains frequently occur. Currently approved later-line therapies target these mutations incompletely with limited clinical benefit. M4205, a kinome-selective KIT inhibitor, was designed to address this high unmet medical need by inhibiting all relevant *KIT* driver and resistance mutations. Compared with imatinib, M4205 shows stronger antitumor activity in preclinical GIST models driven by oncogenic *KIT* driver mutations. M4205 demonstrates clinically relevant efficacy in a range of preclinical GIST models expressing different secondary *KIT* resistance mutations. The kinase selectivity profile of M4205 is superior to the registered standard of care and investigational agents. M4205, now IDRX-42, is currently being investigated in a phase I first-in-human study in participants with GIST.

## Introduction

Gastrointestinal stromal tumors (GIST) are the most common primary mesenchymal tumors of the gastrointestinal tract, representing 0.1% to 3% of all gastrointestinal cancers (1). They arise from the interstitial cells of Cajal (2) and are mainly found within the stomach and small intestine (3). The estimated worldwide incidence is 10 to 20 cases per million per year with 5,000 to 6,000 new cases per year in the United States (4).

The receptor tyrosine kinase (RTK) KIT has been identified as a key oncogenic driver in about 80% of metastatic GIST cases. Primary activating mutations in either the extracellular region (exon 9; 10%) or the juxtamembrane domain (exon 11; 67%) result in ligand-independent constitutive activation of KIT, inducing uncontrolled cell growth and survival (5). Imatinib, a tyrosine kinase inhibitor targeting Bcr–Abl, KIT, and PDGFRA, is approved as a first-line therapy for patients with unresectable metastatic or recurrent GIST (6, 7) and has demonstrated remarkable clinical activity with a median progression-free survival (PFS) of 24 months and a 68% overall response rate (ORR). However, within 12 to 36 months, approximately 50% to 70% patients will progress on imatinib therapy (7, 8). Secondary resistance mutations in *KIT* kinase domains, either in the ATP pocket (exon 13/14) or in the activation loop (exon 17/18), are the most common cause in about 50% to 80% of patients (9, 10). The currently available second- and third-line therapy options, sunitinib and regorafenib, provide rather limited clinical benefit with a median PFS of less than 6 months (11, 12). This is in line with the fact that neither sunitinib nor regorafenib are adequately covering the range of disease-associated mutations of *KIT* at tolerated exposure levels (9, 13). Recently, ripretinib was approved as a fourth-line treatment option for patients with GIST (14). Ripretinib is a switch-control kinase inhibitor designed to block all clinically relevant driver and resistance mutations of *KIT* (15). However, a recent phase III clinical trial comparing the efficacy of ripretinib with sunitinib in second line failed to demonstrate a clinical benefit of ripretinib over sunitinib (16). Avapritinib is a selective type I KIT-mutant inhibitor particularly potent against mutations in the activation loop of *KIT* but lacking activity against mutations in the ATP pocket (17). A recently completed phase III study demonstrated no benefit for avapritinib compared with regorafenib in third- and fourth-line patients (18). In contrast, avapritinib showed an ORR of almost 90% in GIST tumors bearing a D842V activating mutation in *PDGFRA* (19) which is present in about 10% of patients with GIST*.* This impressive clinical activity and manageable safety profile resulted in the line-agnostic approval for patients with GIST harboring *PDGFRA* exon 18 mutations.

Overall, current treatment options for patient with advanced GIST progressing on imatinib are limited by two issues: first, the emergence of resistance mutations in the ATP pocket and in the activation loop of KIT and second, a poor kinome selectivity of second-generation kinase inhibitors resulting in unfavorable safety profiles and leading to dose modifications and drug holidays (20, 21). The development of intra- and intertumoral heterogeneity of KIT resistance mutations under imatinib therapy and insufficient coverage of all relevant mutations by later lines of treatment may explain the variable patient response and overall shorter PFS. Multiple resistance mutations in *KIT* in the same or different lesions have been described for a large proportion of patients with GIST across different cohorts (22–24). Applying advanced next-generation sequencing technologies for detection of *KIT* mutations in plasma-derived ctDNA further confirmed the substantial intrapatient heterogeneity of KIT mutations in patients with later-line GIST (25, 26).

Therefore, a high unmet clinical need remains for novel KIT inhibitors to treat patients with GIST who progress on or are intolerant to imatinib. More recently, novel KIT inhibitors were developed to address this issue, including bezuclastinib (PLX9486), another type I KIT inhibitor active against mutations in the activation loop of *KIT* which is differentiated from avapritinib by its limited brain penetration (27). Bezuclastinib is currently being investigated in combination with sunitinib in a phase III study as second-line treatment in patients with GIST (28). Additionally, the selective KIT-mutant inhibitor AZD3229, which was discovered by AstraZeneca (29) and was licensed as NB003 to NingBo Newbay Technologies, recently started a phase I study in patients with advanced solid tumors, including GIST (30). Upcoming clinical data will show whether these novel drugs overcome the challenges of polyclonal resistance mechanisms and tumor heterogeneity of advanced GIST.

The identification of M4205, a highly kinome-selective KIT inhibitor with broad *KIT*-mutant activity, was recently described in detail (31). Here, we report the preclinical characterization of M4205 as a potent and highly selective inhibitor of disease relevant oncogenic and resistance mutations of *KIT*. Compared with the standard of care (SoC) and investigational agents, M4205 has superior potency against a range of *KIT* mutations and a best-in-class kinase selectivity profile translating into clinically meaningful antitumor activity in a broad panel of GIST xenograft models. Predicted human effective concentrations of M4205 are below inhibitory concentrations for critical off-targets like FLT3 and VEGFR2. The preclinical data strongly support the clinical development of M4205 which is currently being investigated as IDRX-42 in a phase I first-in-human study with promising early efficacy signals across different KIT mutations (32) in participants with advanced (metastatic and/or surgically unresectable) GIST.

## Materials and Methods

### Protein crystallography

**Figure 1.**
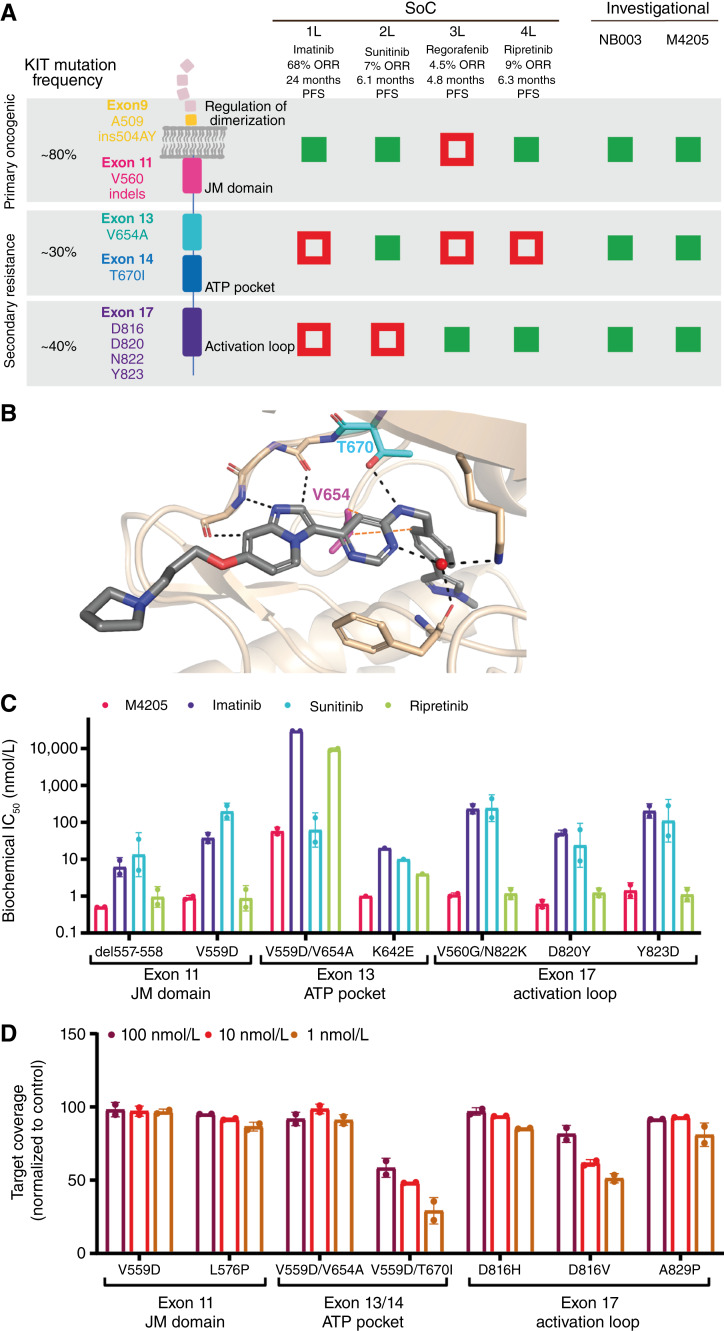
Discovery of M4205, a potent inhibitor of disease-relevant KIT-mutant variants. **A,** Schematic overview of clinically observed mutations in different domains of the RTK KIT and estimated target coverage (green: positive; red: negative) of approved and investigational KIT inhibitors. 1L, first-line; 2L, second-line; 3L, third-line; 4L, fourth-line. **B,** X-ray structures of M4205 (2.1 Å resolution; PDB ID: 7ZW8) in complex with the kinase domain of wild-type KIT. For clarity, only interacting residues are shown including H-bonds and Van der Waals contacts (depicted as dashed black and brown lines, respectively). **C,** Potent biochemical inhibition of clinically relevant KIT mutations in exon 11, exon 13/14, and exon 17 is demonstrated for M4205 compared with approved KIT inhibitors imatinib, sunitinib, and ripretinib. IC_50_ values show mean with SD of two technical replicates. **D,** Cellular target engagement of KIT-mutant forms with M4205 is confirmed in NanoBRET assays. Target coverage is determined by competitive displacement of the NanoBRET tracer with 1, 10, or 100 nmol/L M4205 and is shown as mean with SD of two technical replicates.

The co-crystal structure of M4205 in complex with the kinase domain of wild-type KIT (551-935, delta 694-753-Thr-Ser) was solved at 2.1 Å resolution [Protein Data Bank (PDB) ID: 7ZW8] as described previously. Briefly, 10 mg/mL KIT (551-935, delta 694-753-Thr-Ser) premixed with 2 mmol/L sunitinib was crystallized at 20°C in a buffer containing 9% (w/v) polyethylene glycol 8,000 and 0.1 mol/L HEPES (pH 7.25) in a vapor diffusion experiment. For soaking, a suitable protein crystal was washed three times with the same buffer to remove sunitinib and then M4205 was added as 2.54 mg/mL final concentration. After soaking for 1 week at 20°C, the crystal was transferred into a cryo-buffer with 25% glycerol and flash cryo-cooled in liquid nitrogen. The flash-frozen crystal was used for X-ray diffraction data collection at a temperature of 100 K at the Swiss Light Source. The crystals belong to space group P2_1_2_1_2_1_. Data were processed using the programs autoPROC (RRID: SCR_015748), XDS (RRID: SCR_015652), and AIMLESS (RRID: SCR_015747). The structure was solved by molecular replacement using Phaser (RRID: SCR_014219) and a published KIT crystal structure (PDB ID: 3G0E). Manual model rebuilding was performed in COOT (RRID: SCR_014222) with alternated rounds of structure refinement in BUSTER (version 2.11.8: Global Phasing Ltd., RRID: SCR_015653). Figure 1B was generated based on the KIT•M4076 crystal structure (PDB ID: 7ZW8) with the software tool PyMOL (The PyMOL Molecular Graphics System, version 2.3.0, Schrödinger, LLC, RRID: SCR_000305).

### Biochemical kinase assays

*In vitro* kinase assays with radioactive assay formats were performed at Reaction Biology Corp. according to their standard protocols. The HotSpot kinase profiling service was used for *in vitro* selectivity profiling in single-dose singlicate mode at a concentration of 1 μmol/L against 398 kinases. IC_50_ values for KIT-mutant kinases and for FLT3 and VEGFR2 (*KDR*) were determined with dose–response curves using a 10-dose threefold serial dilution in duplicates. Fitting of dose–response curves and calculation of IC_50_ values were performed with sigmoidal dose–response equations using GraphPad Prism software (RRID: SCR_002798; version 8).

### Cellular target engagement assays

Binding affinities of test compounds to different KIT-mutant proteins in intact living cells were assessed with NanoBRET assays at Reaction Biology Corp. according to their standard protocols. Briefly, respective KIT-mutant NanoLuc fusion vectors were transiently expressed in HEK293 cells (RRID: CVCL_0045). The cells were pretreated with the respective NanoBRET Tracer and then incubated with test compounds at 1, 10, and 100 nmol/L or DMSO for 1 hour. The NanoBRET NanoGlo substrate was added, and BRET signals were measured on an EnVision 2104 Multilabel Reader [PerkinElmer; 450 nm band pass (donor) and 610 nm long pass (acceptor)]. Dasatinib at a concentration of 10 μmol/L was used as reference for all KIT-mutant forms (except 10 μmol/L CEP-701 was used for *KIT*^V559D/T670I^) and subtracted as background. The target coverage of individual KIT-mutant forms by test compounds was determined by normalizing to the DMSO-treated control.

The binding of test compounds to FLT3 in intact living cells was assessed using the FLT3 NanoBRET assay according to the manufacturer’s instructions (Promega). The FLT3 NanoLuc fusion plasmid was transfected into HEK293T cells (RRID: CVCL_0063). After 20 hours, the cells were treated with a 10-dose threefold serial dilution of test compounds and 500 nmol/L tracer K-5 was added. Upon incubation for 2 hours at 37°C, the NanoBRET NanoGlo substrate was added, and BRET signals were determined on an EnVision 2104 Multilabel Reader [PerkinElmer; 450 nm band pass (donor) and 610 nm long pass (acceptor)]. The competition for FLT3 binding by test compounds was referred to the response of no-tracer samples and the values of DMSO-treated controls. Normalization and calculation of apparent dissociation constant (Kd) values were performed with Genedata Screener (RRID: SCR_022506, Genedata).

### Cell lines and culture conditions

GIST430, GIST430/654, and GIST48B (RRID: CVCL_M441) cell lines were obtained under a nonexclusive research license agreement for non-patented material from the Brigham and Women’s Hospital, Harvard Medical School, Boston, MA. GIST430 cells harbor a deletion of amino acids 560 to 576 in exon 11 of KIT and GIST430/654 cells harbor a deletion of amino acids 560 to 576 in exon 11 and a secondary imatinib resistance mutation V654A in exon 13 of KIT, whereas GIST48B cells lack expression of KIT (personal communication Dr. Jonathan A. Fletcher/Brigham and Women’s Hospital; ref. 33). GIST cell lines were cultured in Iscove’s modified Dulbecco’s medium containing 15% FCS and supplemented with 100 nmol/L imatinib for GIST430/654 cells only. The Kasumi-1 cell line (RRID: CVCL_0589) and the MOLM-13 cell line (RRID: CVCL_2119) were acquired from DSMZ and grown in RPMI-1640 medium supplemented with 20% FCS. Mv-4-11 cells (RRID: CVCL_0064) were obtained from ATCC and cultured in Iscove’s modified Dulbecco’s medium containing 10% FCS. THP-1 cells (RRID: CVCL_0006) were obtained from ATCC and cultured in RPMI-1640 medium supplemented with 10% FCS. All cell lines were only used at up to 30 passages and were not cultured longer than 4 months. Quality control analyses including confirmation of the absence of *Mycoplasma* infection, identity testing by short tandem repeat analysis, and confirmation of cell line cross-contamination were regularly performed.

### Cellular signaling assays

Cells were seeded in culture medium supplemented with 15% FCS in 96-well plates with 2.2 × 10^4^ cells (GIST430) or 2.5 × 10^4^ cells (GIST430/654 and Kasumi-1) per well. The following day, when the cells were about 80% confluent, treatment with serial dilutions of test compounds or DMSO was performed in triplicate for 45 minutes. The cells were washed once with TBS and lysed with 90 μL ice-cold lysis buffer (20 mmol/L HEPES pH 7.5, 200 mmol/L NaCl, 1.5 mmol/L MgCl_2_ × 6H_2_O, 0.4 mmol/L EDTA, 1% Triton X-100, 20 mmol/L β-glycerophosphate, 1% Phosphatase-Inhibitor Set II, 0.1% Protease-Inhibitor Cocktail Set III, and 0.01% Benzonase). Cell lysates were then analyzed for levels of P-Y703-KIT in a Luminex-based assay using a mouse total KIT antibody (Abcam, cat #ab111033, RRID: AB_10862477) coupled with Luminex microsphere beads for capturing and a rabbit monoclonal P-Y703-KIT (Cell Signaling Technology, cat #3073, RRID: AB_1147635) or a rabbit monoclonal KIT (Cell Signaling Technology, cat #3074, RRID: AB_1147633) and a PE-labeled donkey anti-rabbit antibody (Jackson ImmunoResearch, cat #711-116-152, RRID: AB_2340599) for detection. Levels of isolated P-Y703-KIT were determined with a Luminex LX200 device (Luminex Corporation) according to the manufacturer’s instructions by counting 100 events per sample.

Cellular levels of ERK1/2 phosphorylation were detected in drug-treated GIST430, GIST430/654, and Kasumi-1 cell lysates using the MSD MULTI-SPOT Biomarker Detection Base Kit Phospho (T202/Y204; T185/Y187)/Total ERK1/2 according to the manufacturer’s instructions (MSD, #15107A-3).

Levels of phosphorylated AKT (S473) were detected in a Luminex-based assay using a mouse total AKT antibody (Millipore, cat #05-591, RRID: AB_309826) coupled with Luminex microsphere beads for capturing and a P-S473-AKT antibody (Cell Signaling Technology, cat #4058, RRID: AB_331168) as well as a PE-labeled donkey anti-rabbit antibody (Jackson ImmunoResearch, cat #711-116-152, RRID: AB_2340599) for detection.

Levels of tyrosine-phosphorylated human macrophage colony–stimulating factor receptor (M-CSF1R) were detected in THP-1 cells in a sandwich ELISA–based assay (R&D systems, #DYC3268-2). Cells were treated with serial dilutions of test compounds in technical duplicates for 25 minutes and then stimulated with 500 ng/mL M-CSF for another 5 minutes. Cell lysates were prepared and levels of phosphorylated CSF1R were determined according to the manufacturer’s instructions using a capture antibody specific for human M-CSF1R and a horseradish peroxidase–conjugated monoclonal antibody specific for phosphorylated tyrosine.

Counts from solvent-treated control cells were set as maximum signal (100%) and compound-treated samples were calculated as the percent of control. Fitting of dose–response curves and calculation of IC_50_ values were performed with nonlinear least squares regression analysis using GraphPad Prism software (RRID: SCR_002798; version 8).

### Cellular viability assays

Cells were seeded in culture medium supplemented with 15% FCS in 96-well plates with 0.1 × 10^4^ cells (GIST48B and Kasumi-1), 0.3 × 10^4^ cells (GIST430), or 0.25 × 10^4^ cells (GIST430/654) per well. The following day, when cells were about 15% confluent, treatment with serial dilutions of test compounds or DMSO was performed in triplicate. After 7 days, cell viability was determined by adding 10 μL resazurin reagent (R&D Systems, #AR002) per well and fluorescence (544 nm excitation/590 nm emission) was measured after 4 to 5 hours. Fluorescent counts from wells containing medium only were subtracted as background from all cell samples. Cell viability levels from solvent-treated control cells were set as maximum signal (100%) and compound-treated samples were calculated as the percent of control. Fitting of dose–response curves and calculation of IC_50_ values were performed with nonlinear least squares regression analysis using GraphPad Prism software (RRID: SCR_002798; version 8).

### Cell line viability screen

A cell line viability screen was performed at Horizon Discovery using 320 cancer cell lines across 18 different tissue types (breast, 24; colorectal, 33; endometrium, 14; esophagus, 7; head and neck, 19; kidney, 7; leukemia, 15; liver, 26; lung, 56; lymphoma, 31; myeloma, 10; ovary, 25; pancreas, 14; prostate, 3; skin, 15; soft tissue, 2; stomach, 17; and urinary, 2). Cells were thawed from a liquid nitrogen–preserved state and once cells divided at their expected doubling times, the cells were seeded in black 384-well tissue culture–treated plates. At the time of treatment, ATP levels of cells which did not receive treatment were measured using CellTiter-Glo 2.0 (Promega) to determine a time zero state. The cells were treated for 6 days with a threefold serial dilution (10 μmol/L to 1.52 nmol/L) of either M4205 or ripretinib. After treatment, endpoint analysis was performed using CellTiter-Glo 2.0 and concentrations leading to a half-maximal growth inhibition normalized to time zero state (GI_50_) were calculated using proprietary software of Horizon Discovery.

### *In vivo* efficacy and pharmacokinetic/pharmacodynamic studies

*In vivo* efficacy data were generated in mice with different xenograft models for GIST and acute myeloid leukemia (AML) tumors. An overview of the different *in vivo* models is provided in Supplementary Table S1, including mutational information and sensitivity to clinical compounds. Studies with cell line–derived xenograft (CDX) models GIST430 and GIST430/654 and GIST patient–derived xenografts (PDX) GS11342 were performed in house at the healthcare business of Merck KGaA. The study designs and animal usage were approved by local animal welfare authorities (for internal studies Regierungspräsidium Darmstadt, Germany; protocol registration numbers DA4/Anz.1014, DA4/Anz.1040, DA4/1017, and DA4/226). Female H2d Rag2 mice (7–8 weeks old) were obtained from Taconic Denmark. Tumor cell lines GIST430 and GIST430/654 were subcutaneously inoculated once in the right flank of H2D Rag2 female mice with 5 million cells in a volume of 100 μL. The PDX models GS11342 (*KIT* mutation WKV557fs) and GS11328 (*KIT* mutation WK557del) were subcutaneously transplanted as tumor fragments of 1 to 2 mm size (*N* = 1 per mouse). When tumors reached a volume of 140 to 230 mm^3^, the animals were randomized, and treatment was initiated to ensure that all treatment groups had the same mean starting tumor volume. Each treatment group consisted of 10 mice, each with one tumor; thus, each experiment represented a biological replicate of 10. In concordance with German animal welfare regulations, most *in vivo* studies were performed once, relying on the statistical power based on a high animal number as opposed to repeat experiments.

Studies with GIST PDX GS11331 (mutations in *KIT* p.WK557del and V654A) and GS5108 (mutations in *KIT* p.WK557del and Y823D) were performed by Crown Bioscience. Animal welfare was compliant with the US Department of Agriculture’s Animal Welfare Act (9 CFR Parts 1, 2, and 3) as applicable and was covered by the Institutional Animal Care and Use Committee–approved animal protocol #EB17-030. Female NOD/SCID mice were obtained from The Jackson Laboratory. A total of 100 μL of cell suspension (50–100,000 cells) in cold RPMI medium with Cultrex ECM was subcutaneously inoculated into the rear flank of mice. All animals were randomly allocated to the four different study groups. Randomization was performed in the Study Log software with a randomization range of 170 to 190 mm^3^.

Studies with the AML CDX model Kasumi-1 (mutation in *KIT* exon 17 N822K) were performed by Chempartner. Animal welfare was compliant with local animal welfare regulations. Female NOD/SCID mice were obtained from Beijing Vital River Lab Animal Technology Co. A total of 1 billion cells suspended in 200 μL Matrigel were injected into the rear flank of mice. All animals were randomized into treatment groups (*n* = 10 per treatment arm, randomized from 15 mice per arm).

Treatment with the respective compound occurred by oral gavage. Compound formulation and dosing are described in Supplementary Table S2. Body weight of mice was measured daily. Tumor length (L) and width (W) were measured twice weekly by calipers. The tumor volume was calculated using the formula (L × W^2^)/2.

*In vivo* efficacy was tested for statistical significance using two-way ANOVA with GraphPad Prism software (RRID: SCR_002798; version 8), applying an alpha of 5%. Statistical significance in *in vivo* graphs is indicated by the use of letters, in which the same letters denote groups that are not statistically different from one another, whereas different letters signify statistically significant differences between groups.

Treatment group:control group effect (T/C) ratios were calculated to assess the effect of a treatment on tumor growth at the end of each study following preclinical RECIST criteria (34). The following formulas were used: for % T/C (for mean values > 0): [(end tumor volume treatment − start tumor volume treatment)/(end tumor volume control − start tumor volume control)] × 100) and % T/C (for mean value ≤ 0, i.e., regression): [(end tumor volume treatment − start tumor volume treatment)/start tumor volume treatment] × 100.

Histopathology was performed in tissue fixed in 4% formalin and processed for hematoxylin and eosin−stained sections. For clinical chemistry, serum was prepared from coagulated blood and processed in an ADVIA 1800 Autoanalyzer. For hematology, blood samples were diluted with anti-coagulant EDTA and processed in an ADVIA 120 Autoanalyzer.

Levels of total KIT and autophosphorylated KIT at tyrosine 703 (Y703) were detected as pharmacodynamic biomarkers. Tumors were excised and 50 to 100 mg tissue was lysed with a Precellys 24 homogenizer (Bertin Technologies). Total protein concentrations were determined using the BCA Protein Assay Kit (Thermo Fisher Scientific, #23225), and 50 μg of the lysate was analyzed in technical triplicates using the Luminex-based assay described above. Counts for P-Y703-KIT were normalized to counts for total KIT for each technical sample. The average of the normalized P-Y703-KIT level from the vehicle-treated probes (*n* = 5) was set as 100% control and counts of probes treated with M4205 were calculated as the percent of control. The quantitative determination of M4205 in mouse plasma was done by high-performance liquid chromatography coupled with tandem mass spectrometry.

### Human dose prediction

Efficacious exposures were determined in different mouse xenograft models using noncompartment analyses of mouse pharmacokinetic (PK) data. Observed mouse PK data after single and repeated administration of three different dose levels were best described by applying a two-compartment PK model with linear clearance (CL), using the software Phoenix WinNonlin 6.4 v.8.3.3.33 (RRID: SCR_024504).

Mean human exposure at the steady state following repeated dosing of M4205 at the proposed efficacious dose range was estimated using predicted human PK parameters, assuming linear PK and a one-compartment model. To integrate uncertainty on human PK prediction, Monte Carlo simulations were run in R software (version 3.5.1, RRID: SCR_003005) using 1000 iterations.

### Data availability

Atomic coordinates and structure factors of the co-crystal structure of M4205 in complex with KIT have been deposited in the PDB (www.pdb.org) with the accession code 7ZW8. Additional data generated in this study are available upon request from the corresponding author.

## Results

### M4205 is a potent and highly selective inhibitor of KIT-mutant variants

M4205 was discovered by a medicinal chemistry optimization campaign using an imidazopyridine scaffold which was originally identified in a high-throughput screen of the European Lead Factor library using a biochemical assay with the KIT V654A enzyme (31). The optimization strategy leading to the identification of M4205, recently described in detail (31), focused on broad and potent coverage of disease-relevant mutations of KIT, offering superiority over approved SoC drugs for patients with unresectable metastatic or recurrent GIST (Fig. 1A). The crystal structure of M4205 in complex with the kinase domain of KIT was determined (Fig. 1B), rationalizing its activity against KIT mutations in the ATP pocket by direct interactions with V654 and the gatekeeper T670. Although *in vitro* assays with truncated kinase forms are not entirely reflective of KIT kinase activity in a physiologic cellular system, biochemical potency data indicated that M4205 offers broader KIT-mutant coverage of M4205 compared with SoC drugs (imatinib, sunitinib, and ripretinib; Fig. 1C). When comparing biochemical inhibition of KIT mutants in exon 11, exon 13, and exon 17 with highest frequency in patients with GIST according to the American Association for Cancer Research Genie database (Supplementary Table S3; ref. 35), M4205 shows superior activity versus sunitinib for activation loop mutants (exon 17) and is significantly more potent than ripretinib against ATP pocket mutants (exon 13). Remarkably, M4205 also demonstrated a more potent inhibition of oncogenic driver mutation in exon 11 in comparison with the first-line treatment imatinib. Superior activity of M4205 compared with clinical and investigational KIT inhibitors including avapritinib and NB003, formerly known as AZD3229 (29), was further confirmed in a broader biochemical KIT-mutant kinase panel (Supplementary Table S4). Cellular target engagement assays showed high binding affinity of M4205 to various KIT mutants in exon 11, exon 13, and exon 17 (Fig. 1D). At concentrations down to 1 nmol/L, M4205 achieved over 80% target coverage for KIT exon 11 mutants (V559D and L576P), KIT exon 13 mutant V654A, and two KIT exon 17 mutants (D816H and A829P). A higher M4205 concentration of 100 nmol/L resulted in at least 50% target coverage of KIT exon 13 mutation T670I and over 80% target coverage of KIT exon 17 mutation D816V.

**Figure 2.**
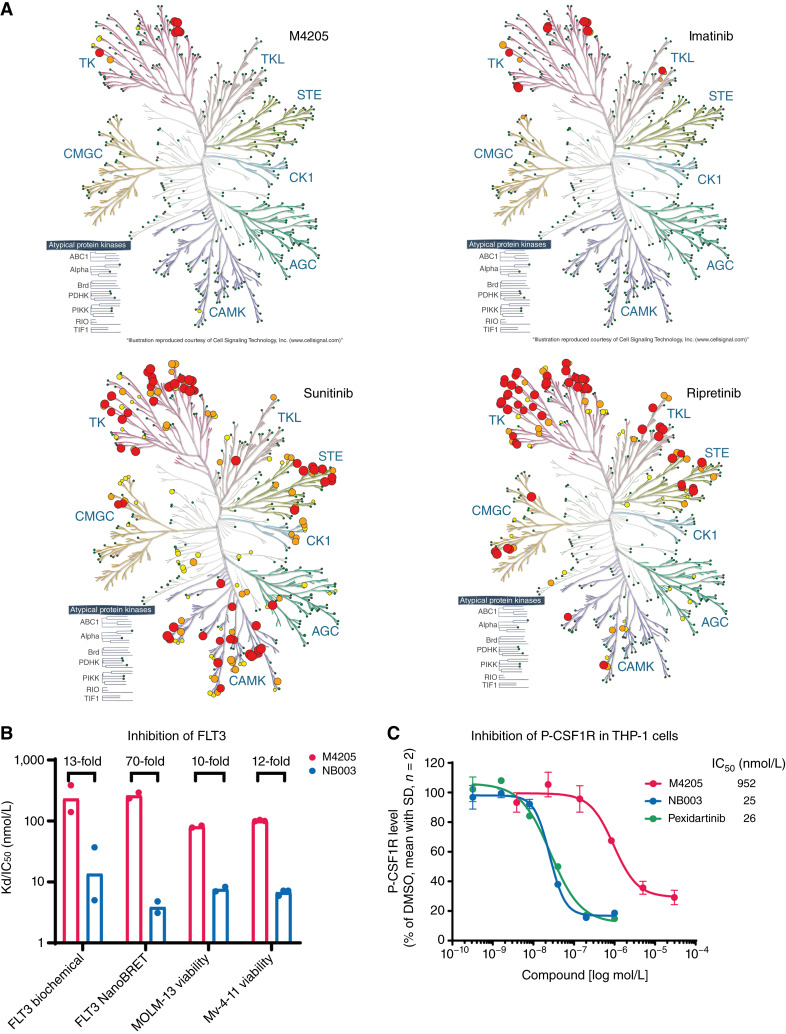
Kinome selectivity of M4205. **A,** Selectivity of M4205 in the HotSpot kinase profiling panel (Reaction Biology) compared with approved and investigational KIT inhibitors at 1 μmol/L. The dots are scaled to the inhibition of the corresponding kinase and color coded (green <30%, yellow >30% and <60%, orange >60% and <80%, and red >80%). Kinase tree illustration reproduced courtesy of Cell Signaling Technology Inc. **B,** M4205 is significantly less potent against FLT3 compared with NB003 as confirmed in biochemical, cellular target engagement, and cellular viability assays in FLT3–ITD–mutated cell lines MOLM-13 and Mv-4-11. Apparent dissociation constant (Kd) and IC_50_ values are derived from at least two independent experiments and shown as mean with SD. **C,** M4205 is less potent in inhibiting CSF1R autophosphorylation in THP-1 cells compared with pexidartinib and NB003 upon compound treatment for 30 minutes. A representative study is shown, and results were confirmed in two independent biological experiments.

Kinome selectivity of M4205 was assessed in a large *in vitro* panel of 398 different kinases. A test concentration of 1 μmol/L M4205 inhibited KIT and five additional related RTKs [PDGFRA, PDGFRB, CSF1R (*FMS*), FLT3, and LCK] by more than 80%, and respective IC_50_ values are reported elsewhere (31). PDGFRA, PDGFRB, CSF1R, FLT3, and KIT are members of the class III RTK family and share highest sequence homology which likely contributes to the observed biochemical activity of M4205 against these kinases. Compared with approved KIT inhibitors imatinib, sunitinib, regorafenib, and ripretinib, M4205 has a superior kinase selectivity profile whereas the investigational KIT inhibitor NB003, formerly known as AZD3229 (29), shows similar selectivity (Fig. 2A; Supplementary Fig. S1A). However, compared with NB003, M4205 is significantly less potent against FLT3 in biochemical and cellular target engagement as well as viability assays in FLT3–ITD–mutated cell lines (Fig. 2B). FLT3 is viewed as a critical off-target kinase based on potential overlapping toxicities with inhibition of wild-type KIT in hematopoietic stem cells (36) and is thus monitored as a selectivity measure during the optimization of M4205 (31). Biochemical kinase testing has identified CSF1R as another potential off target for M4205. However, M4205 showed only weak cellular inhibition of CSF1R autophosphorylation with an IC_50_ of 952 nmol/L in THP-1 cells, a human monocytic cell line expressing high CSF1R levels (Fig. 2C). Pexidartinib, a clinically approved CSF1R inhibitor for the treatment of tenosynovial giant cell tumors (37), served as a positive control and is significantly more potent with an IC_50_ of 26 nmol/L. Of note, the investigational KIT inhibitor NB003, formerly known as AZD3229 (29), is also a cellularly potent inhibitor of CSF1R with an IC_50_ of 25 nmol/L.

Remarkably, M4205 at concentrations up to 10 μmol/L did not induce any inhibition of VEGFR2 (*KDR*; Supplementary Fig. S1B). VEGFR2 is another highly homologous but important off-target kinase within the family of RTKs that could cause dose-limiting toxicities, i.e., hypertension, as observed for sunitinib (38) and regorafenib (39).

### M4205 is more potent than SoC treatments across primary and secondary KIT mutations *in vitro*

**Figure 3.**
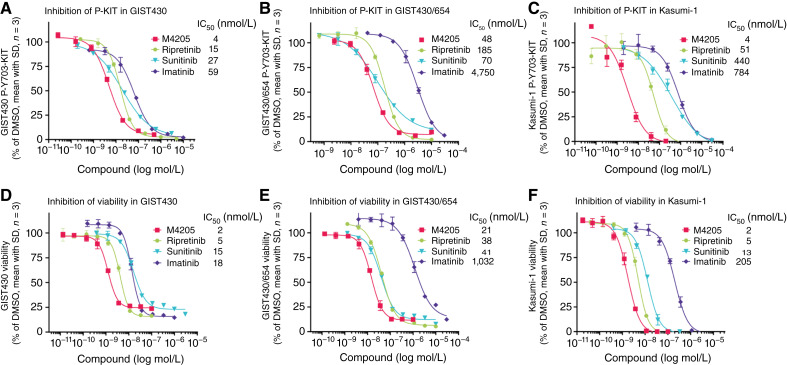
Cellular activity of M4205 compared with clinically approved KIT inhibitors. **A**–**C,** M4205 is more potent than imatinib, sunitinib, and ripretinib in inhibiting autophosphorylation (Y703) of KIT in GIST430 (*KIT* exon 11 del560-576), GIST430/654 (*KIT* exon 11 del560-576/exon 13 V654A), and Kasumi-1 (*KIT* exon 17 N822K) cells upon compound treatment for 45 minutes. **D–F,** Potent on-target inhibition translated into reduction of cell viability of GIST430, GIST430/654, and Kasumi-1 cells treated with respective compound concentrations for 7 days. Shown are individual experiments with technical triplicates and IC_50_ values are derived from two independent biological repeats (Supplementary Tables S5 and S6).

To confirm the broad and potent coverage of disease-relevant KIT mutations indicated by biochemical assays and in cell lines with transgenic overexpression, cellular activity of M4205 was investigated in cancer cell lines expressing disease-relevant endogenous KIT mutations. Inhibition of KIT autophosphorylation at Y703 was assessed as the most proximal pharmacodynamic marker and was further supported by measuring the inhibition of downstream ERK1/2 and AKT phosphorylation. M4205 inhibited KIT autophosphorylation with an IC_50_ of 4 nmol/L in GIST430 cells bearing an oncogenic driver mutation in exon 11 of KIT (del560-576) and was at least 10-fold more potent compared with imatinib and three to fourfold more potent than sunitinib and ripretinib (Fig. 3A). In GIST430/654 cells expressing the imatinib resistance mutation V654A in KIT exon 13 combined with a deletion in exon 11 (del560-576), M4205 induced potent inhibition of KIT autophosphorylation with an IC_50_ of 48 nmol/L. Sunitinib showed similar potency, whereas ripretinib was threefold less potent and imatinib was barely active (Fig. 3B). In the AML cell line Kasumi-1 bearing an N822K mutation in KIT exon 17, M4205 inhibited KIT autophosphorylation with an IC_50_ of 4 nmol/L, whereas SoC drugs were significantly less potent (ripretinib 12-fold, sunitinib 110-fold, and imatinib 190-fold; Fig. 3C). Similarly, in all three cell lines, M4205 showed superior cellular activity compared with the other approved KIT inhibitors regorafenib and avapritinib. The investigational drug NB003 seemed to have comparable KIT inhibition potency with M4205 (Supplementary Table S5). The strong inhibition of KIT autophosphorylation by M4205 in disease-relevant GIST cell lines translated to a potent reduction of downstream receptor signaling. Activation of P-ERK1/2 and P-AKT, key oncogenic drivers of cellular proliferation and survival pathways, was reduced within the same potency range as KIT inhibition (Supplementary Fig. S2).

In addition, M4205 treatment led to a strong reduction of cell viability in KIT-mutated cell lines GIST430 (Fig. 3D), GIST430/654 (Fig. 3E), and Kasumi-1 cells (Fig. 3F), with IC_50_ values in the same potency range as those for inhibiting KIT autophosphorylation and downstream signaling pathways. M4205 was consistently more potent than imatinib, sunitinib, ripretinib, regorafenib, and avapritinib (Supplementary Table S6). In contrast, almost no effect was observed for M4205 in the KIT-independent GIST48B cell line (IC_50_ of 16 μmol/L), whereas ripretinib was more potent with an IC_50_ of 715 nmol/L which could be attributed to the inferior kinase selectivity profile.

The cellular selectivity profile of M4205 was evaluated in a comprehensive viability screen of 320 cancer cell lines representing 18 different tissues (Supplementary Fig. S3A). Growth of cancer cell lines usually depends on unique but different oncogenic properties and the activity profile of a compound can be used to assess its specificity. Sensitivity to M4205 with GI_50_ values below 100 nmol/L was only found in six cell lines characterized by driver alterations in kinases targeted by M4205. Activity of M4205 was confirmed in the *KIT*-mutated AML cell line Kasumi-1 (as shown in Fig. 3F), which was the only cell line with a known *KIT* driver mutation presented in the panel. Inhibition of viability of FTL3–ITD–positive AML cell lines MOLM-13 and MV-4-11 (as shown in Fig. 2B) was verified as well. In line with the biochemical activity of M4205 against PDGFRA (31), three additional cell lines bearing an oncogenic PDGFRA pathway alteration showed inhibition of viability with GI_50_ values below 100 nmol/L (Supplementary Fig. S3A and S3B). Inhibition of viability with GI_50_ potencies in the range of 100 nmol/L to 1 μmol/L M4205 was only observed in 10 additional cell lines, further supporting the strong selectivity profile of M4205. In contrast, ripretinib reduced cell viability with GI_50_ values below 1 μmol/L in 159 (50%) of the tested cell lines (Supplementary Fig. S3A).

### M4205 demonstrates dose-dependent pharmacodynamic modulation of KIT activation *in vivo*

**Figure 4.**
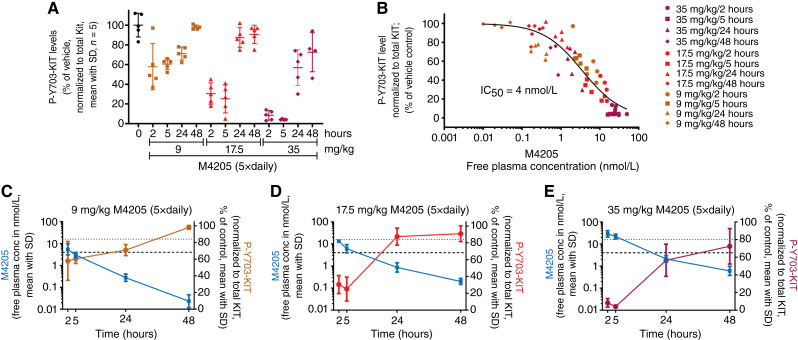
Dose- and exposure-dependent inhibition of KIT autophosphorylation with M4205 in the GIST430/654 model. **A,** Tumor-bearing mice were treated daily with three different doses of M4205 for 5 days, and inhibition of KIT autophosphorylation compared with untreated controls was determined at different time points after last treatment. **B,** Free plasma concentrations of M4205 were plotted against the effect of KIT inhibition which revealed an *in vivo* IC_50_ of 4 nmol/L. **C–E,** Inhibition of KIT autophosphorylation is correlated with free plasma concentration over time for the three dosage groups (dashed line: 4 nmol/L free *in vivo* IC_50_; dotted line: 16 nmol/L free cellular IC_50_).

To test the PK and pharmacodynamic activity of M4205 *in vivo* at the steady state, mice bearing GIST430/654 CDXs were treated with different doses once daily for 5 days. Plasma concentration of M4205 as well as levels of autophosphorylated KIT in tumor tissue was determined at different time points after the last treatment at study end. A dose-dependent reduction of phosphorylated KIT in tumor tissue was observed over time (Fig. 4A) which translated also into dose-dependent inhibition of downstream ERK1/2 phosphorylation (Supplementary Fig. S4A), in line with the cellular data. Free plasma concentration of M4205 was calculated considering the fraction unbound in mice (0.903%) and plotted versus levels of phosphorylated KIT. The calculated IC_50_ of 4 nmol/L for inhibition of KIT phosphorylation was in good alignment with the cellular free IC_50_ of 16 nmol/L (Fig. 3B: 48 nmol/L total, corrected for protein binding to 15% FCS in cell culture medium) in the GIST430/654 model (Fig. 4B). Increase in the exposure of M4205 is dose linear, and a free concentration in the range of the free IC_50_ correlates with strong inhibition of KIT autophosphorylation (Fig. 4C–E).

Dose-dependent inhibition of KIT autophosphorylation in response to either single or repeated treatment with M4205 was confirmed in other GIST *in vivo* tumor models bearing different activating and resistance mutations of *KIT* (Supplementary Fig. S4B–S4D).

### M4205 inhibits *in vivo* tumor growth in models with primary activating *KIT* mutations as well as ATP-binding pocket and activation loop mutations

At the onset of continuous treatment studies in mice, tolerability and safety of M4205 was assessed. M4205 was well tolerated at doses up to 35 mg/kg when administered daily without any signs of toxicity such as apathy, changes in body score, histopathologic findings, or body weight loss whereas dosages of 150 mg/kg once daily led to body weight loss within 1 week (Supplementary Fig. S5A). Antitumor growth assessment in the same study indicated that the maximum effect in a relevant GIST CDX was already reached at 35 mg/kg daily (Supplementary Fig. S5B). Follow-on studies demonstrated that doses of 25 mg/kg daily were well tolerated even upon daily administration for 160 days (Supplementary Fig. S5C). Histopathologic analysis of a range of potential target organs indicated minimal dose-dependent effects on hematopoietic cells at dosages of 35 mg/kg daily and higher (Supplementary Fig. S5D; Supplementary Table S7) which could be confirmed by hematologic assessment of whole-blood samples (Supplementary Fig. S5D; Supplementary Table S8). Clinical chemistry indicated spontaneous changes in bilirubin and aspartate aminotransferase (ASAT) at dosages of 35 mg/kg daily and higher which were considered non-treatment related (Supplementary Fig. S5D; Supplementary Table S9). This led to the conclusion that M4205 was well tolerated and safe at dosages up to 35 mg/kg daily.

**Figure 5.**
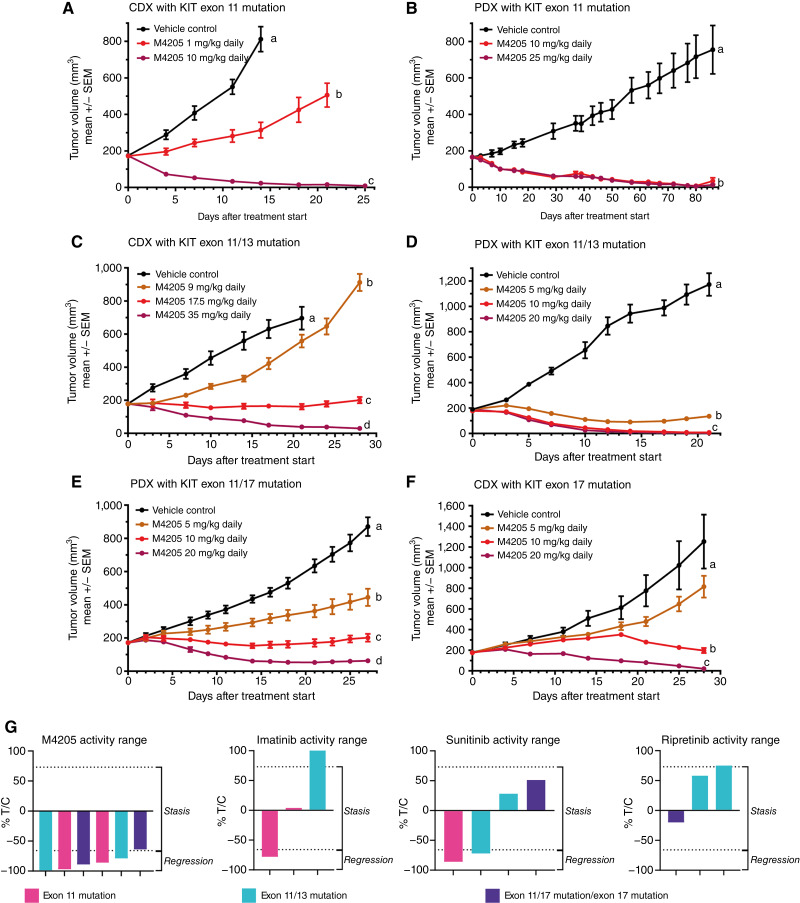
*In vivo* antitumor activity of M4205 in xenograft models expressing oncogenic *KIT* mutations. Tumor bearing mice were treated daily with M4205 by oral gavage at doses indicated (10 mice per group). Body weight of mice was measured daily, and tumor growth was followed twice weekly. **A,** Exon 11–mutant GIST CDX model GIST430 (del560-576). **B,** Exon 11–mutant GIST PDX model (GS11342 and WKV557fs). **C,** Exon 11–mutant (del560-576) and exon 13–mutant (V654A) GIST CDX model GIST430/654. **D,** Exon 11–mutant (WK557del) and exon 13–mutant (V654A) GIST PDX model GS11331. **E,** Exon 11–mutant (WK557del) and exon 17–mutant (Y823D) GIST PDX model GS5108. **F,** Exon 17–mutant (N822K) AML CDX model Kasumi-1. Different letters within the graph indicate statistical significance (*P* < 0.05) according to two-way ANOVA analysis on log-transformed data. **G,** Treatment effects (%) for these different studies were plotted as T/C ratios. According to preclinical RECIST criteria, T/C ratios between 73% and −66% indicate tumor stasis and T/C ratios below −66% indicate tumor regression.

The antitumor activity of M4205 *in vivo* was evaluated in models with endogenous *KIT* mutations and compared with relevant clinically approved KIT inhibitors. M4205 led to complete regressions at a dosage of 10 mg/kg daily in a cell line–derived and a patient-derived tumor model with a primary activating mutation in exon 11 (Fig. 5A and B). These tumor models responded well to imatinib at a dose equivalent in exposure to the clinically used dose as expected, yet notably the response to M4205 was even superior to imatinib in the CDX model (Fig. 5G; Supplementary Fig. S6A and S6B). Next, *in vivo* models with an imatinib resistance mutation in the ATP-binding pocket (V654A) were tested to assess whether M4205 was able to inhibit growth in these imatinib-resistant tumor models. In a V654A-mutant CDX model, we observed dose-dependent tumor growth inhibition in response to M4205 treatment, inducing stable disease at a dose of 17.5 mg/kg and tumor shrinkage at 35 mg/kg (Fig. 5C and G). In a V654A-mutant PDX model, M4205 could even induce complete responses at doses of 10 and 20 mg/kg (Fig. 5D and G). To confirm the expected response of SoC compounds (Fig. 1A), we also treated the V654A-mutant CDX model with imatinib, sunitinib, ripretinib, avapritinib, and regorafenib at doses which were exposure-equivalent to the respective clinical dose. As expected, because of the V654A resistance mutation, GIST430/654 tumors did not respond to imatinib (Supplementary Fig. S6C), whereas sunitinib induced tumor stasis (Supplementary Fig. S6D) but did not inhibit tumor growth as effectively as M4205 (Fig. 5C). When treated with ripretinib, avapritinib, or regorafenib, tumors continued to grow under treatment (Supplementary Fig. S6D–S6F), confirming that none of these agents are active on GIST tumors with a V654A resistance mutation. In the V654A-mutant PDX model, sunitinib was effective and induced tumor stasis, whereas ripretinib, which had to be discontinued prematurely because of poor tolerability, did not inhibit tumor growth during the treatment period (Supplementary Fig. S6G).

To assess the *in vivo* efficacy of M4205 in tumor models with resistance mutations in the activation loop of *KIT*, a PDX model with a *KIT* mutation in exon 17 (Y823D) was tested. Similar to the V654A-mutant model, M4205 induced dose-dependent tumor growth inhibition with tumor shrinkage at the highest dosage of 20 mg/kg daily (Fig. 5E). The same model did not respond to sunitinib treatment but responded well to ripretinib treatment (Supplementary Fig. S5H). Additionally, M4205 showed dose-dependent antitumor efficacy in an AML model with a *KIT* driver mutation in exon 17 (Kasumi-1 and N822K) with regression observed at the highest dosage of 20 mg/kg daily (Fig. 5F).

Overall, comparing the respective *in vivo* efficacy by plotting % T/C values from the individual experiments, the broad tumor activity of M4205 across different KIT mutations becomes apparent whereas the SoC drugs imatinib, sunitinib, and ripretinib only cover some mutations at doses equivalent to clinical doses (Fig. 5G).

**Figure 6.**
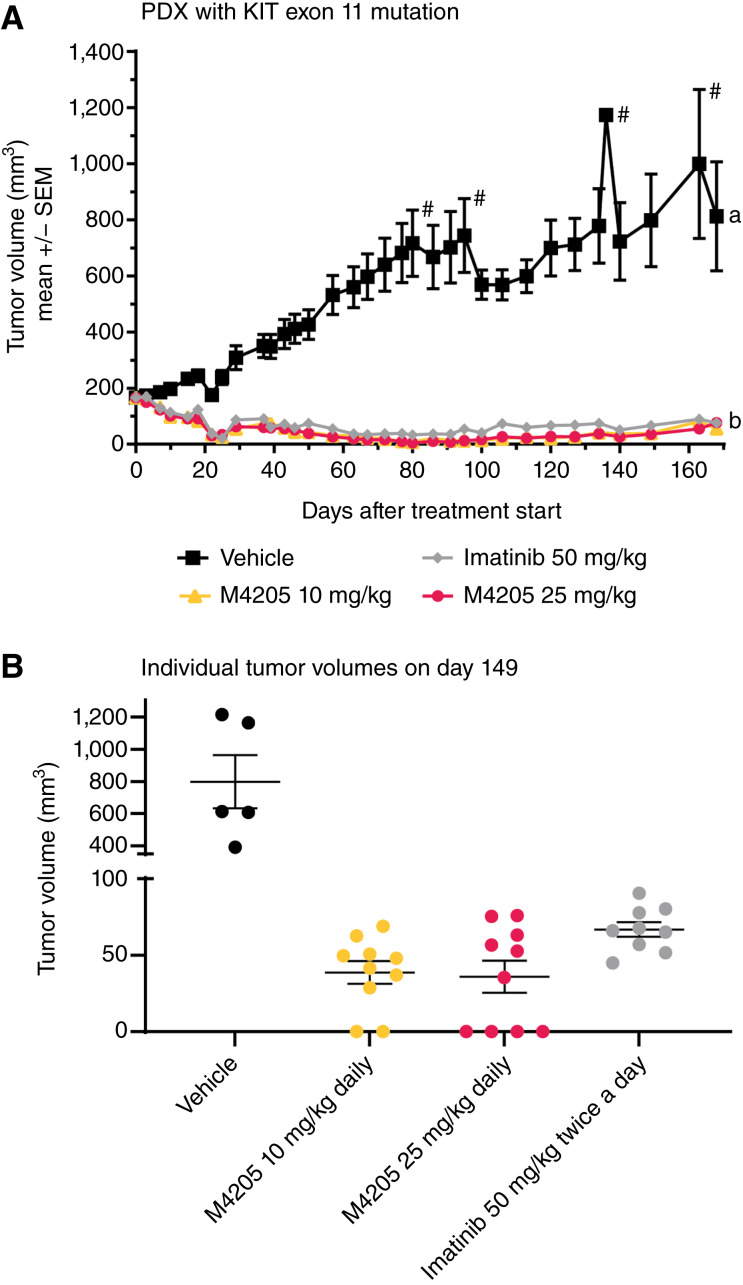
Long-lasting antitumor effect under treatment with M4205 in a GIST PDX model with a *KIT* exon 11 mutation. Mice bearing a GIST PDX model with a *KIT* exon11 mutation (GS11328 WK557del) were treated continuously for 160 days once daily with M4205 or twice daily with imatinib at the indicated doses. *N* = 10 per group at beginning of the study. **A,** ANOVA statistical testing confirmed a strong and highly significant tumor growth inhibition in all treatment groups compared with the control group as indicated by the letters. Hashtags indicated time points when mice from the vehicle control group had to be sacrificed because of tumor volume. **B,** Dot plot with individual tumor volumes for each mouse after 149 days indicates a trend for increased full regressions with M4205 whereas tumors in the imatinib group remained palpable.

Considering the broad activity of M4205 on different *KIT* mutations, it was explored whether any treatment resistance under *in vivo* therapy could be observed. Mice bearing a PDX model with an oncogenic *KIT* exon 11 mutation were continuously treated daily with either 10 or 25 mg/kg M4205 or with imatinib at a dose equivalent to the respective clinical dose over a period of 160 days (Fig. 6A). All three treatments led to significant tumor regression relative to the vehicle control throughout the entire study duration without signs of regrowth. There was a trend for more lasting full regressions in the M4205 groups compared with imatinib (Fig. 6B), but the overall difference between M4205 and imatinib was not significant. Longer follow-up was not possible because mice started to age, nearing the end of their natural life span.

### Prediction of the human efficacious dose range

Prediction of human PK parameters for M4205 was based on *in vitro* to *in vivo* extrapolation, using human hepatocytes for plasma clearance and the Øie–Tozer model for volume of distribution integrating *in vivo* PK data of rat, monkey, and dog (31). Plasma CL was estimated as 0.20 L/hours/kg and the volume of distribution was calculated as 9.5 L/kg, resulting in a predicted terminal half-life of M4205 of approximately 33 hours. The efficacious dose range of M4205 was defined as 10 to 35 mg/kg daily considering the antitumor activity in various xenograft mouse models expressing different KIT mutations (Fig. 5). A two-compartment mouse PK model with linear elimination described the observed exposure in mice best (Supplementary Fig. S7A and S7B) and determined a free efficacious AUC_0-24h_ of 52 to 180 ng × hours/mL which corresponds to a human total exposure of 1,800 to 6,300 ng × hours/mL. By incorporating the above mentioned predicted human PK parameters and the free exposure range in the preclinical efficacy models, a human efficacious dose range of 40 to 140 mg daily was predicted assuming a one-compartment model. Additionally, when considering uncertainties for estimation of human PK parameters, i.e., the human CL prediction, the biologically efficacious human dose is estimated to be in the range of 24 to 260 mg daily within the 80% confidence interval (Supplementary Fig. S7C). Corresponding median-free average concentration values at the steady state were 4 to 16 nmol/L, which is well in line with the free IC_50_ value of 4 nmol/L determined for inhibition of KIT autophosphorylation in the GIST430/654 model (Fig. 4B). Notably, the predicted free efficacious concentration of M4205 is approximately five- to 16-fold lower than IC_50_ values for FLT3 inhibition, indicating a sufficient safety margin at a daily dose of 260 mg M4205 (Supplementary Table S7).

## Discussion

The establishment of imatinib as first-line therapy for patients with unresectable metastatic or recurrent GIST was considered a breakthrough for a disease with inherent resistance to chemo- and radiotherapy (40, 41). As a potent inhibitor of the RTK KIT, an oncogenic driver in about 80% of metastatic GIST, imatinib’s authorization also initiated a new era for the development of targeted therapies against cancers with molecularly defined driver alterations. Yet, despite the long PFS rate of 24 months, secondary on-target resistance mutations emerge in about 50% to 80% of the patients progressing on imatinib (9, 10). Imatinib-induced resistance mutations occur either in the ATP pocket or in the activation loop of *KIT* and show broad intra- and intertumoral heterogeneity, including heterogeneity between lesions within one patient (42, 43).

Sunitinib and regorafenib are currently approved second- and third-line therapies for patients with GIST progressing on imatinib. However, neither inhibitor achieves sufficiently broad coverage of secondary *KIT* mutations reflected in limited response rates and PFS. Although sunitinib is potent against mutations in the ATP pocket of KIT in exons 13 and 14, but rather inactive against mutations in the activation loop of KIT in exon 17 (9, 33), preclinical and clinical data showed an opposite activity profile for regorafenib against secondary KIT resistance mutations (13). Furthermore, both drugs suffer from a poor kinase selectivity profile, leading to hand-foot syndrome related to multitargeted tyrosine kinase inhibition (44, 45), overlapping hematopoietic toxicities due to dual inhibition of FLT3 and KIT (36, 46) and high-grade hypertension linked to VEGFR2 inhibition (38, 47). The limited tolerability related to poor kinase selectivity results in dose reduction or disruption potentially restricting clinical activity and affects quality of life of patients. Therefore, a high unmet need remains for potent KIT inhibitors with activity on a broad range of KIT driver and resistance mutations while exhibiting high kinome selectivity for KIT.

The switch control kinase inhibitor ripretinib has been designed as a KIT inhibitor with a broad coverage of disease-relevant mutations because of its dual mode of action regulating both the kinase switch pocket and the activation loop (15). Ripretinib has been approved as fourth-line treatment for advanced GIST based on 9% ORR and 6.3 months median PFS in a placebo randomized phase III study (14). Although initial preclinical data suggested activity of ripretinib against primary oncogenic mutations as well as secondary resistance mutations in the ATP pocket and activation loop of *KIT* (15), a phase III trial for second-line therapy of patients with advanced GIST failed to meet its primary PFS endpoint when compared with sunitinib (16). Notably, our preclinical *in vivo* studies dosing ripretinib with the murine equivalent to the human dose demonstrate limited activity in GIST xenograft models bearing V654A, the most frequent ATP pocket mutation in *KIT*, further reinforcing the need for selective KIT inhibitors with potent activity against the broad spectrum of disease-relevant mutations of KIT.

Several additional novel KIT inhibitors including bezuclastinib (28), NB003 (29), and THE-630 (48, 49) have recently entered early clinical development for advanced GIST, and their safety profiles and efficacy remain to be seen. Theseus Pharmaceuticals lately announced that the maximal tolerated dose of THE-630 was expected to provide exposure below the estimated target levels, leading to the discontinuation of the THE-630 phase I/II trial. Additionally, based on the narrow activity on KIT mutations (bezuclastinib) and suboptimal off-target profiles (NB003), we assume that a significant unmet need for KIT kinase–selective inhibitors with broad KIT mutation coverage remains.

M4205 is specifically designed to target primary oncogenic and secondary resistance mutations of KIT while maintaining high kinome selectivity, especially against related RTKs like FLT3 and VEGFR2, as evidenced by biochemical and cellular kinome assays (31). At the same time, M4205 achieved broad activity across different disease-relevant KIT mutations in biochemical and cellular studies. In cell lines expressing either primary or secondary KIT mutations, M4205 was more potent in blocking cellular KIT signaling compared with all tested SoC drugs, translating to superior viability inhibition. Furthermore, M4205 induced strong antitumor activity, including regressions, across different disease-relevant *in vivo* xenograft models with primary as well as ATP-binding pocket and A loop mutations in KIT, whereas sunitinib or ripretinib only achieved activity in models with secondary resistance mutations in exon 13 or 17, respectively. In line with these preclinical findings, phase I data for M4205 (now IDRX-42) show early efficacy signals across a range of *KIT* mutations in patients with GIST (32).

While NB003 also achieved broad and potent coverage of KIT mutations, it is significantly more active against the critical off-target FLT3 compared with M4205. Whether this off-target activity will lead to enhanced hematopoietic toxicity of NB003 remains to be seen in ongoing clinical testing. The broad KIT-mutant coverage of M4205 was also confirmed in a recent study in GIST PDX and CDX models (50). M4205 demonstrated superior efficacy compared with clinically relevant KIT inhibitors, associated with decreased mitotic activity and antiproliferative effects. Notably, one model with a KIT exon 13 mutation showed myxoid degeneration, which is indicative of a clinical response to KIT inhibitors in patients with GIST. Based on the strong antitumor activity of M4205 in GIST xenograft models with primary oncogenic KIT mutations in exon 11 (this study) and exon 9 (50), which was superior compared with the current first-line treatment imatinib, it is tempting to speculate whether M4205 could even be considered as a first-line therapy option given the significantly lower risk for the occurrence of on-target resistance mutations. In line with this observation, M4205 (now IDRX-42) is currently being tested in a cohort of first-line patients with GIST (32).

The broad activity of M4205 may imply extended duration of response in patients by preventing the development of resistance. However, this is challenging to assess preclinically due to life span limitations of *in vivo* animal studies and lack of models reflecting the disease heterogeneity in patients (22, 23). The absence of tumor growth recurrence in a model bearing a primary activating mutation of *KIT* in exon 11 after long-term treatment with M4205 for up to 160 days is encouraging, yet no resistance against imatinib was observed either despite being well described in patients. Thus, although the totality of data for M4205 indicates broad activity across KIT mutations and therefore potential effectiveness in patients with heterogeneous GIST tumor burden, the potential emergence of novel resistance mechanisms cannot be entirely ruled out and ongoing studies in patients will provide more insights (32). The favorable safety and tolerability features of M4205 described in this article and in ongoing clinical studies (32) suggest potential for combination approaches with M4205 in the future, yet the broad activity against disease-relevant activating and resistance mutations of KIT and early clinical data imply monotherapy activity of M4205 even in early treatment lines.

In addition to the broad and potent coverage of different disease-relevant KIT mutations, a high kinome selectivity profile is considered critical to achieve a sufficient on-target exposure level. The remarkable kinase selectivity of M4205 has been confirmed in biochemical and cellular kinase assays (31) and is further evidenced by good tolerability of M4205 at dosages tested in murine antitumor activity studies and by a clean profile in a broad cell line screen. Based on the excellent PK/PD correlation and the identification of a consistent active dose range across all xenograft efficacy studies, we were able to predict human efficacious concentrations of M4205. Additionally, uncertainties in human PK parameters were incorporated, enabling us to predict a human efficacious dose range of 24 to 260 mg daily within an 80% confidence interval. Encouragingly, estimated efficacious exposure levels of M4205 have a more than five to 16-fold safety margin toward the effective dosage in mice.

In conclusion, the excellent preclinical characteristics and early clinical data of M4205 indicate its potential for a best-in-class new treatment option for patients with advanced GIST.

## Supplementary Material

Supplementary Figure S1 Kinome Selectivity

Supplementary Figure S2 Cellular P-ERK1/2 and P-AKT inhibition

Supplementary Figure S3 Cell line viability screen

Supplementary Figure S4 In vivo P-KIT and P-ERK1/2 inhibition

Supplementary Figure S5 Tolerability in mice

Supplementary Figure S6 In vivo efficacy of SoC drugs

Supplementary Figure S7 Mouse PK and human dose prediction

Supplementary Table S1 Animal model information

Supplementary Table S2 In vivo formulation and administration

Supplementary Table S3 GENIE KIT mutation frequency

Supplementary Table S4 Biochemical inhibition of KIT variants

Supplementary Table S5 Cellular P-KIT inhibition

Supplementary Table S6 Cellular viability inhibition

Supplementary Table S7 Mouse Histopathology

Supplementary Table S8 Mouse Hematology

Supplementary Table S9 Mouse Clinical Chemistry

Supplementary Table S10 Split of M4205 concentration and FLT3 inhibition
