# Supplementary material for: M4205 (IDRX-42) Is a Highly Selective and Potent Inhibitor of Relevant Oncogenic Driver and Resistance Variants of KIT in Cancer
Source: Mol Cancer Ther. 2025 Feb 28;24(7):1040–53. doi: 10.1158/1535-7163.MCT-24-0699 (PMC12214875; doi:10.1158/1535-7163.MCT-24-0699)
Supplement: Supplementary Figure S1 — Kinome Selectivity [file mct-24-0699_supplementary_figure_s1_suppsf1.pdf]

Supplementary Figure S1

A

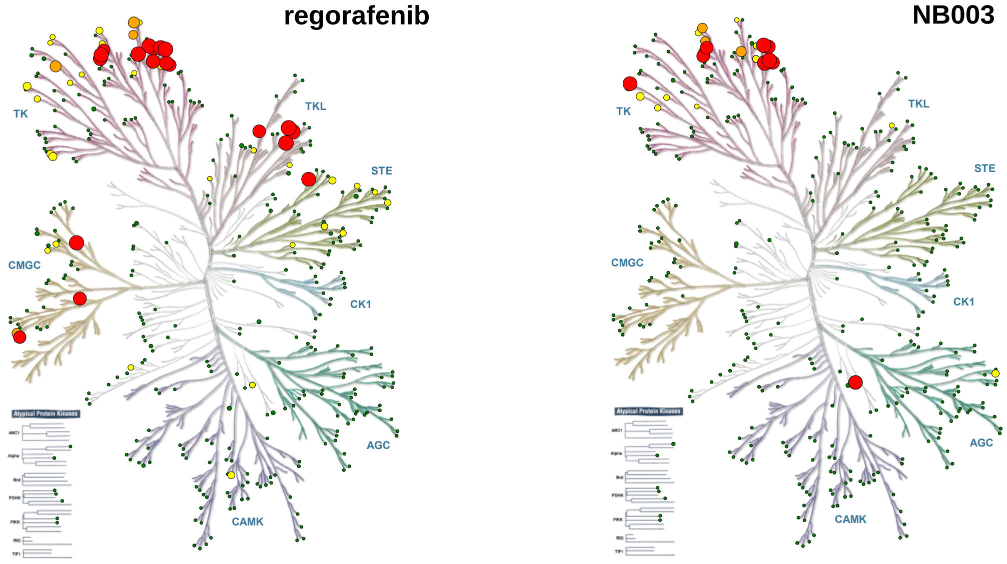

B

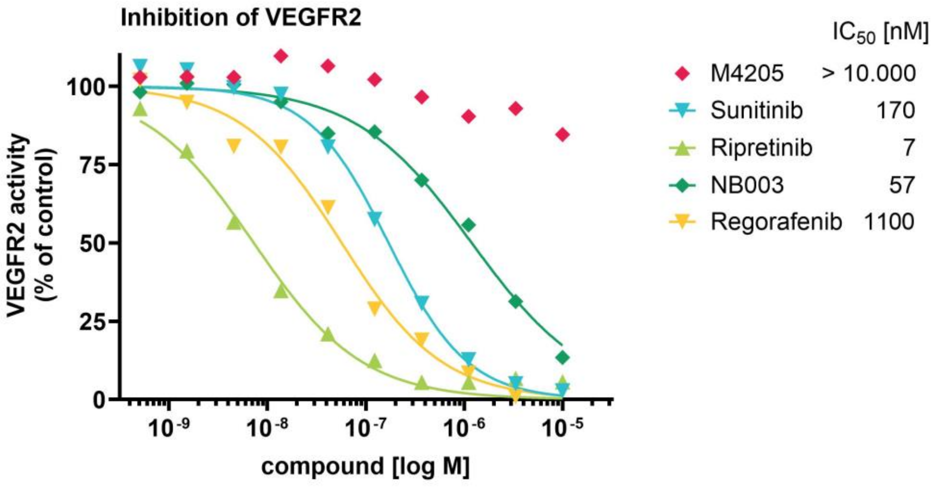

Supplementary Figure S1: **Kinome selectivity.** (A) Selectivity of regorafenib and NB003 in HotSpot kinase profiling panel (Reaction Biology) at 1 µM. The dots are scaled to the inhibition of the corresponding kinase and color coded (green <30%, yellow >30% and <60%, orange >60% and <80% and red >80%). Kinase tree illustration reproduced courtesy of Cell Signaling Technology Inc. (B) Biochemical VEGFR2 (*KDR*) activity in response to treatment with M4205, sunitinib, ripretinib, regorafenib, and NB003.
