# Supplementary material for: M4205 (IDRX-42) Is a Highly Selective and Potent Inhibitor of Relevant Oncogenic Driver and Resistance Variants of KIT in Cancer
Source: Mol Cancer Ther. 2025 Feb 28;24(7):1040–53. doi: 10.1158/1535-7163.MCT-24-0699 (PMC12214875; doi:10.1158/1535-7163.MCT-24-0699)
Supplement: Supplementary Figure S2 — Cellular P-ERK1/2 and P-AKT inhibition [file mct-24-0699_supplementary_figure_s2_suppsf2.pdf]

Supplementary Figure S2

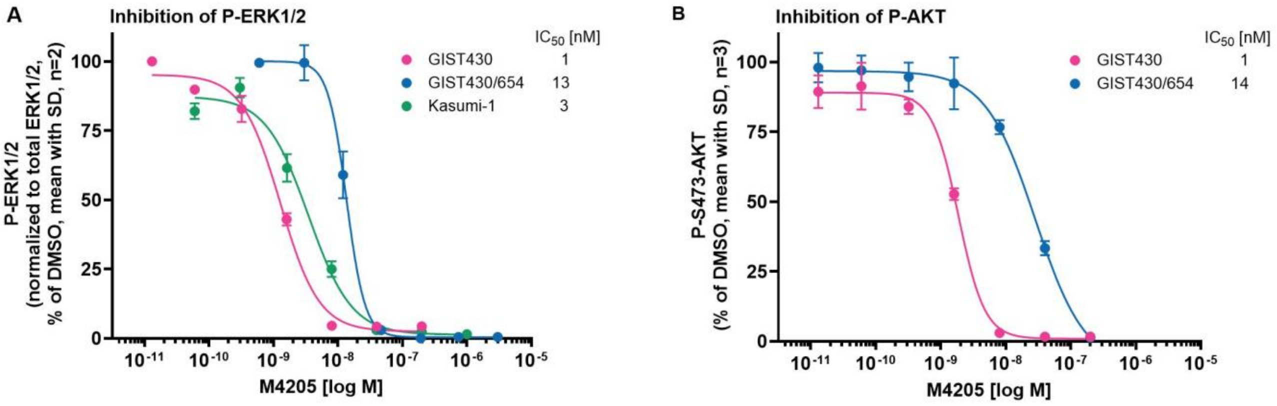

Supplementary Figure S2: **Cellular inhibition of P-ERK1/2 and P-AKT with M4205.** (A)Treatment of KIT mutant cell lines GIST430, GIST430/654 and Kasumi-1 with M4205 for 45 minutes reduced P-T202/Y204-ERK1 / P-T185/Y187-ERK2 level in a dose-dependent manner. (B) Treatment of KIT mutant cell lines GIST430 and GIST430/654 with M4205 for 45 minutes reduced P-AKT level in a dose-dependent manner. Shown are individual experiments with technical duplicates. Signals for P-AKT were below detection level in Kasumi-1 cells.
