# Supplementary material for: M4205 (IDRX-42) Is a Highly Selective and Potent Inhibitor of Relevant Oncogenic Driver and Resistance Variants of KIT in Cancer
Source: Mol Cancer Ther. 2025 Feb 28;24(7):1040–53. doi: 10.1158/1535-7163.MCT-24-0699 (PMC12214875; doi:10.1158/1535-7163.MCT-24-0699)
Supplement: Supplementary Figure S3 — Cell line viability screen [file mct-24-0699_supplementary_figure_s3_suppsf3.pdf]

Supplementary Figure S3

A

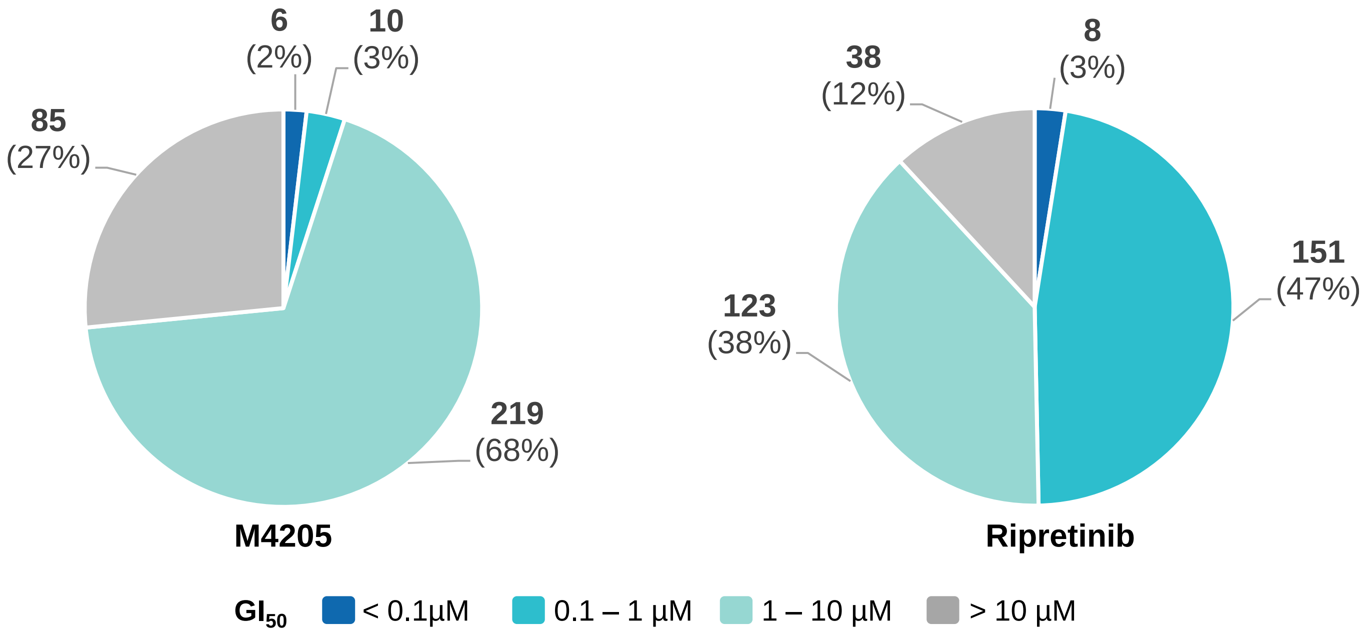

Cell line	Driver Alteration	M4205 GI_{50} [nM]	Ripretinib GI_{50} [nM]
Kasumi-1	KIT N822K	2	1
MOLM-13	FLT3-ITD	7	9
MV-4-11	FLT3-ITD	10	25
EOL-1	FIP1L1-PDGFRA fusion	0.037	0.052
NCI-H1703	PDGFRA amplification	13	41
G-402	PDGFRA overexpression	23	52

B

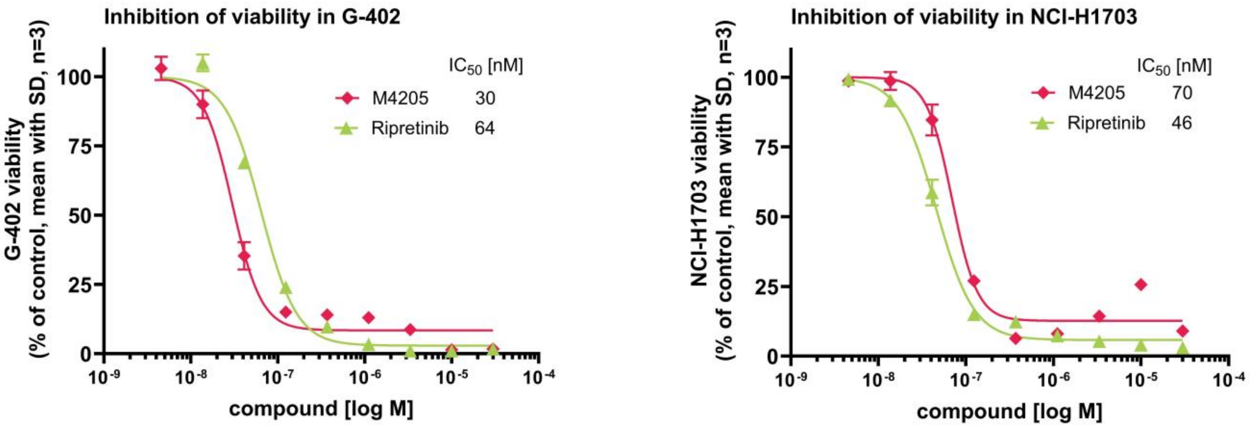

Supplementary Figure S3: **Viability screen of 320 cancer cell lines with M4205 and ripretinib.** (A) Cells were treated with a 3-fold serial dilution (10 µM to 1.52 nM) of M4205 or ripretinib for 6 days. Cellular viability was measured using CellTiter-Glo 2.0 (Promega) at the time of treatment (time zero) and after 6 days. GI_{50} values were calculated by normalization of results to time zero values. The table presents GI_{50} values for M4205 and ripretinib for the six cell lines showing GI_{50} values below 100 nM in response to M4205. (B) Dose-dependent inhibition of viability in response to treatment with M4205 and ripretinib for 6 days was confirmed in G-402 and NCI-H1703 cell lines expressing PDGFRA alterations using a Resazurin-based viability read-out.
