# Supplementary material for: M4205 (IDRX-42) Is a Highly Selective and Potent Inhibitor of Relevant Oncogenic Driver and Resistance Variants of KIT in Cancer
Source: Mol Cancer Ther. 2025 Feb 28;24(7):1040–53. doi: 10.1158/1535-7163.MCT-24-0699 (PMC12214875; doi:10.1158/1535-7163.MCT-24-0699)
Supplement: Supplementary Figure S4 — In vivo P-KIT and P-ERK1/2 inhibition [file mct-24-0699_supplementary_figure_s4_suppsf4.pdf]

Supplementary Figure S4

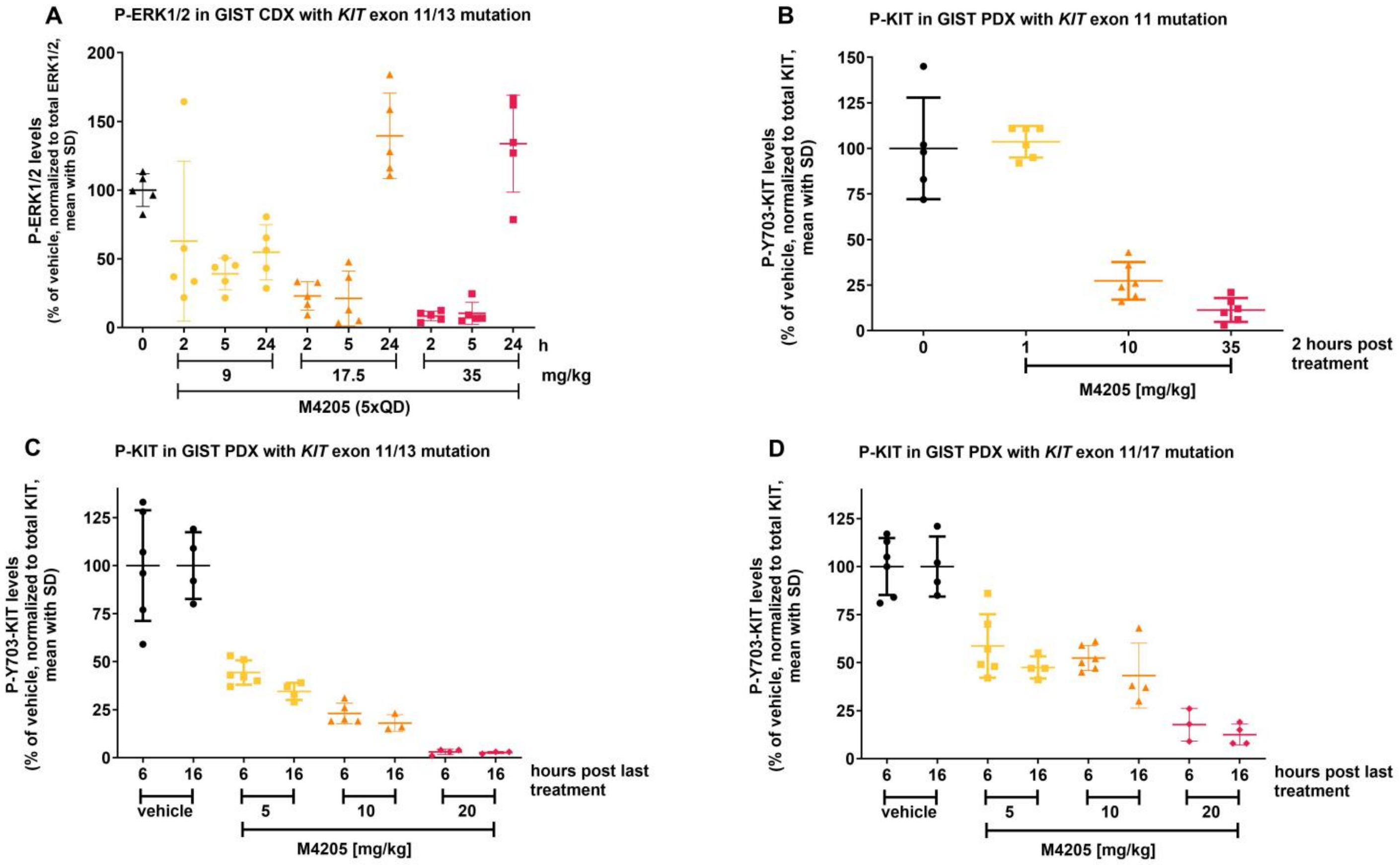

Supplementary Figure S4: **Inhibition of KIT and ERK phosphorylation in GIST tumor models treated with M4205.** (A) Mice bearing tumors of GIST CDX GIST430/654 (*KIT* exon 11 del560-576 / exon 13 V654A) were treated daily with three different doses of M4205 for 5 days and inhibition of ERK1/2 phosphorylation compared to untreated controls was determined at 2, 5 and 24 hours post last dose.(B) Mice bearing tumors of GIST PDX GS1143 (*KIT* exon 11 mutation: WKV557fs) were treated once with three different doses of M4205 and inhibition of KIT autophosphorylation compared to untreated controls was determined at 2 hours post last dose. (C) Mice bearing tumors of GIST PDX GS11331 (*KIT* exon 11 mutation WK557del and exon 13 mutant V654A) were treated with three different doses of M4205 daily for 21 days and inhibition of KIT autophosphorylation compared to untreated controls was determined at 6 and 16 hours post last dose. (D) Mice bearing tumors of GIST PDX GS5108 (*KIT* exon 11 mutation WK557del and exon 17 mutant Y823D) were treated with three different doses of M4205 daily for 21 days and inhibition of KIT autophosphorylation compared to untreated controls was determined at 6 and 16 hours post last dose.
