# Supplementary material for: M4205 (IDRX-42) Is a Highly Selective and Potent Inhibitor of Relevant Oncogenic Driver and Resistance Variants of KIT in Cancer
Source: Mol Cancer Ther. 2025 Feb 28;24(7):1040–53. doi: 10.1158/1535-7163.MCT-24-0699 (PMC12214875; doi:10.1158/1535-7163.MCT-24-0699)
Supplement: Supplementary Figure S5 — Tolerability in mice [file mct-24-0699_supplementary_figure_s5_suppsf5.pdf]

# Supplementary Figure S5

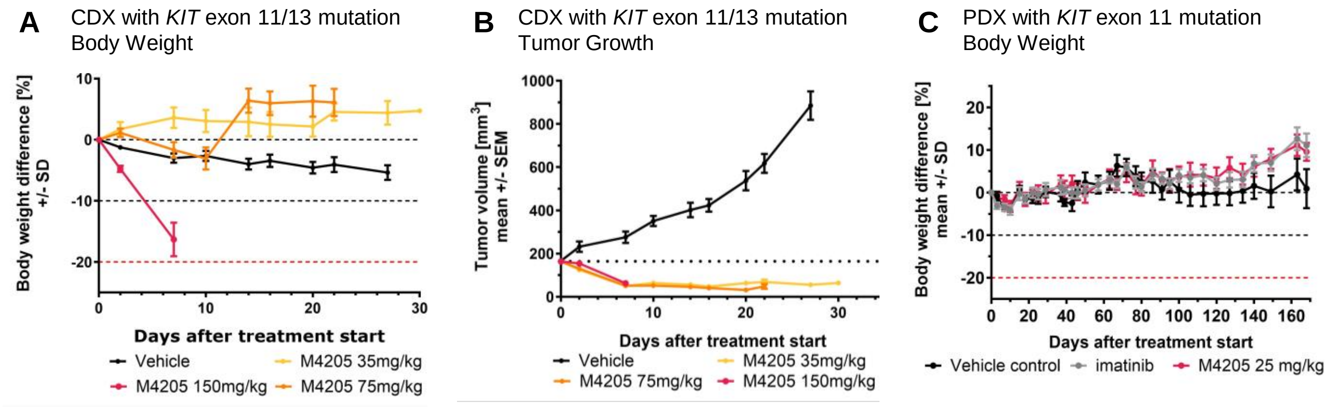


**D**

| | Finding | Control | 17.5 mg/kg | 35 mg/kg | 75 mg/kg | 150 mg/kg |
|---|---|---|---|---|---|---|
| **Histo-pathology** | Spleen: extramedullary hematopoiesis | none | none | minimal | moderate | moderate |
| | Bone marrow: cellularity decrease | none | none | minimal | moderate | moderate |
| | Lung: foamy macrophage | none | none | none | none | minimal |
| | Kidney: hypertrophy | none | none | none | mild | moderate |
| | GI: ulceration | none | none | none | mild | moderate |
| **Hematology** | Cell Count decrease | none | NA | Dose-dependent decrease of: Red Blood Cell, Hematocrit, Hematoglobin, Leukocytes | | NA |
| **Clinical Chemistry** | Bilirubin, ASAT | none | NA | Increase, likely not TR | none | NA |
| | ASAT | none | NA | none | Increase, likely not TR | NA |

Supplementary Figure S5: **Tolerability and safety of M4205 in mice.** (A) Body weight difference relative to the start of treatment. A dosage of 35 mg/kg QD of M4205 was tolerated well when administered daily for 31d to mice bearing the GIST430/654 CDX model with *KIT* exon 11/13 mutation. At a dosage of 75 mg/kg QD, diarrhea and modest body weight loss was observed while a dosage of 150 mg/kg QD led to body weight of 20% within a week. N=10 per group. (B) The anti-tumor effect in the same study indicated that the minimal dose to achieve the maximal effect is 35 mg/kg QD M4205 or less. N=10 per group. (C) In subsequent studies doses up to 25 mg/kg QD were used. These were well tolerated throughout various studies in mice bearing different tumor models, even when treated for 160 days daily as shown in mice bearing a PDX model with a *KIT* exon 11 mutation. N=10 per group. (D) Summary of histopathology, Clinical Chemistry, Hematology, described in detail in Supplementary Tables S7-S9. Sample size N=3-5 per group. TR: Treatment Related, GI: Gastrointestinal, NA sample not available.
