# Supplementary material for: M4205 (IDRX-42) Is a Highly Selective and Potent Inhibitor of Relevant Oncogenic Driver and Resistance Variants of KIT in Cancer
Source: Mol Cancer Ther. 2025 Feb 28;24(7):1040–53. doi: 10.1158/1535-7163.MCT-24-0699 (PMC12214875; doi:10.1158/1535-7163.MCT-24-0699)
Supplement: Supplementary Figure S6 — In vivo efficacy of SoC drugs [file mct-24-0699_supplementary_figure_s6_suppsf6.pdf]

Supplementary Figure S6

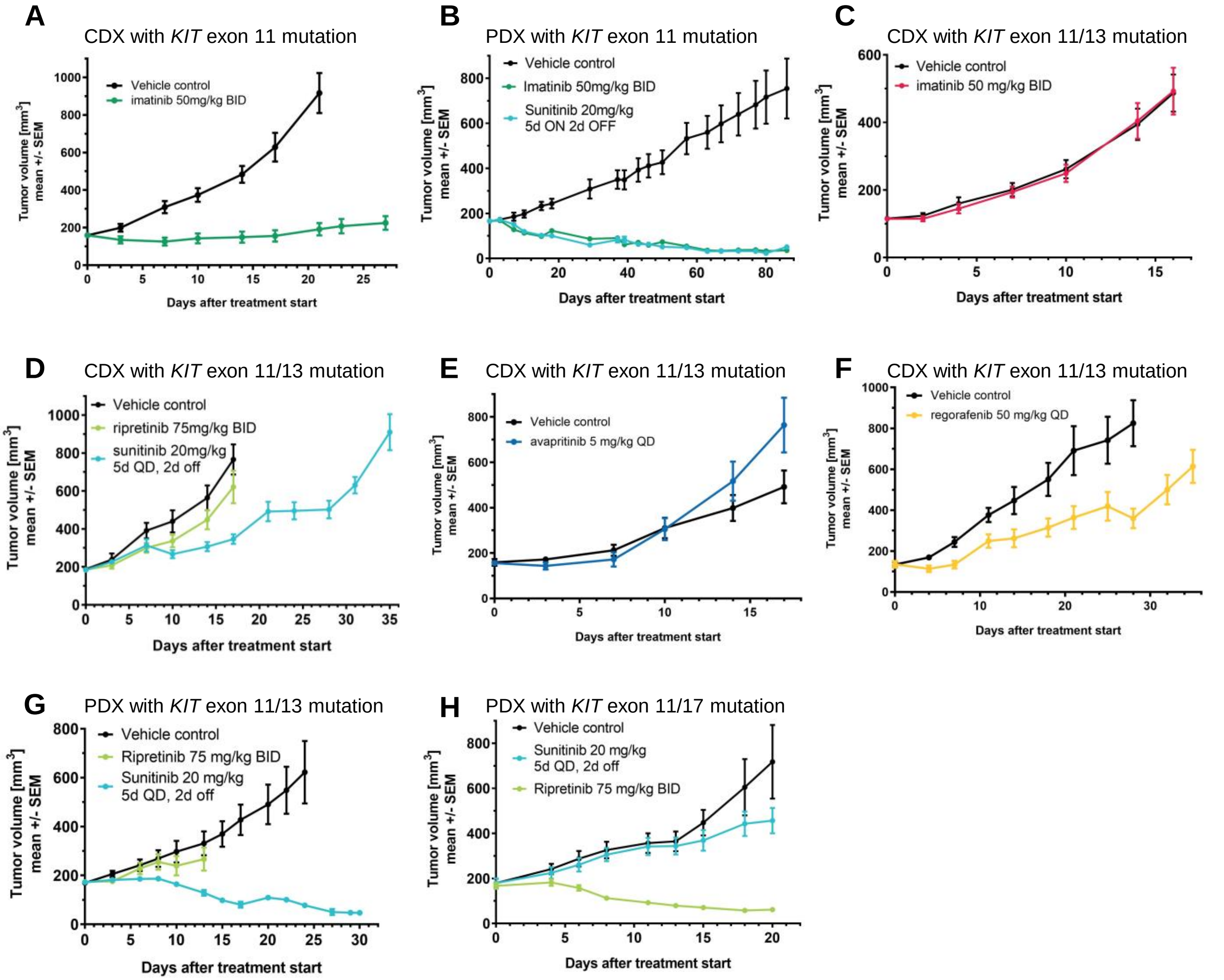

Supplementary Figure S6: **In vivo efficacy curves for SoC and other clinical KIT inhibitors.** Tumor-bearing mice were treated daily (QD) or bi-daily (BID) with indicated inhibitors at doses equivalent to the human efficacious exposure or with respective vehicle control. (A) Exon 11 mutant GIST CDX model GIST430 with *KIT* exon 11 mutation (del560-576). (B) PDX model GS11342 with *KIT* exon 11 mutation (WKV557fs). (C)-(F) CDX model GIST430/654 with *KIT* exon 11 mutation (del560-576) and exon 13 mutation (V654A). (G) PDX model GS11331 with *KIT* exon 11 mutation (WK557del) and exon 13 mutation (V654A). (H) PDX model GS5108 with *KIT* exon 11 deletion (WK557del) and exon 17 resistance mutation (Y823D).
