# Supplementary material for: M4205 (IDRX-42) Is a Highly Selective and Potent Inhibitor of Relevant Oncogenic Driver and Resistance Variants of KIT in Cancer
Source: Mol Cancer Ther. 2025 Feb 28;24(7):1040–53. doi: 10.1158/1535-7163.MCT-24-0699 (PMC12214875; doi:10.1158/1535-7163.MCT-24-0699)
Supplement: Supplementary Figure S7 — Mouse PK and human dose prediction [file mct-24-0699_supplementary_figure_s7_suppsf7.pdf]

Supplementary Figure S7

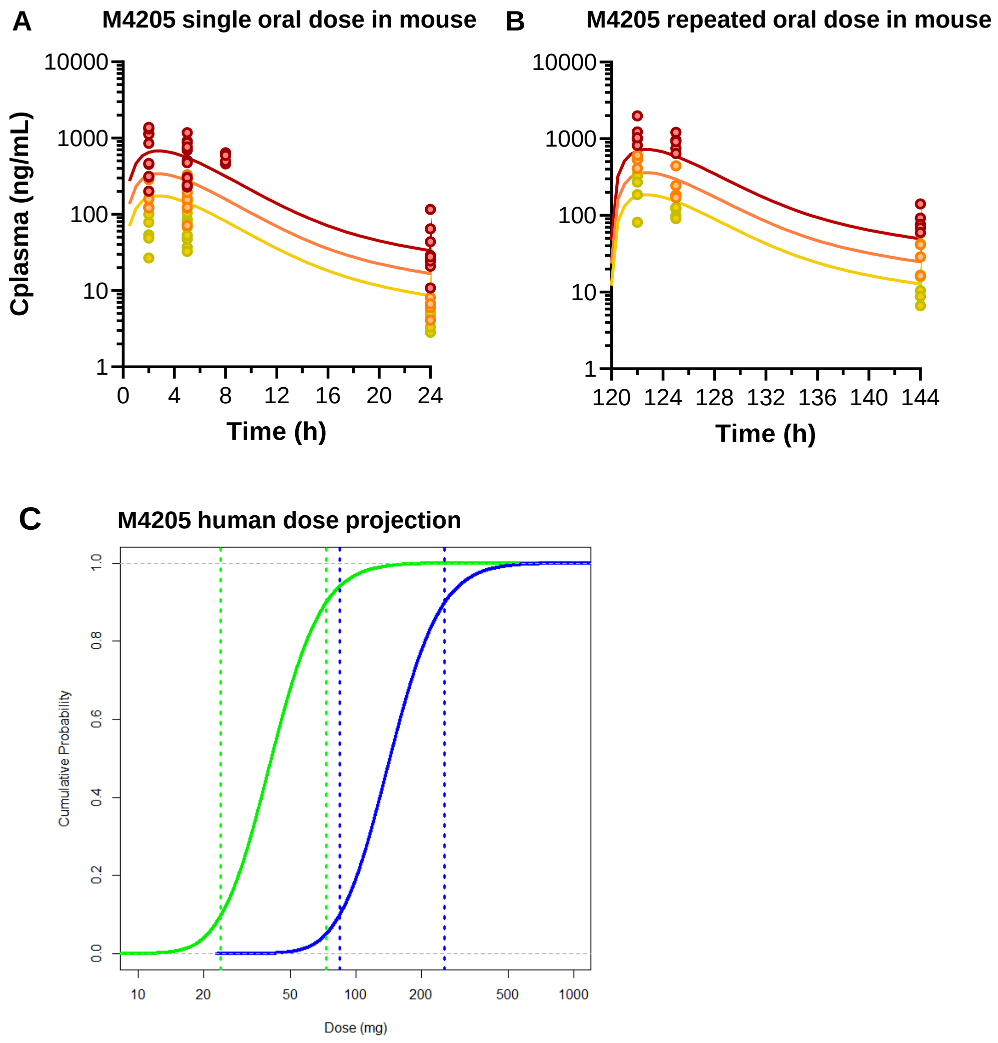

Supplementary Figure S7: **Simulated and observed plasma PK profiles in mouse and human dose prediction for M4205.** Overlay of simulated (─) and observed (●) plasma level of M4205 in mouse after (A) single oral dose and (B) repeated oral dose (5xQD]. (●) 9 mg/kg QD, (●) 17.5 mg/kg QD, (●) 35 mg/kg QD. (C) Monto Carlo simulations for the human dose of M4205 predicted with a confidence interval of 80% (dotted lines) considering the lower (10mg/kg; green line) and higher (35 mg/kg; blue line) range for antitumor efficacy in mouse xenograft models as well as uncertainty on predicted human PK parameters.
