# Supplementary material for: M4205 (IDRX-42) Is a Highly Selective and Potent Inhibitor of Relevant Oncogenic Driver and Resistance Variants of KIT in Cancer
Source: Mol Cancer Ther. 2025 Feb 28;24(7):1040–53. doi: 10.1158/1535-7163.MCT-24-0699 (PMC12214875; doi:10.1158/1535-7163.MCT-24-0699)
Supplement: Supplementary Table S1 — Animal model information [file mct-24-0699_supplementary_table_s1_supps1.pdf]

Supplementary Table S1

Animal Model Information

	Origin	Mutation	Sensitivity to imatinib and sunitinib
GIST430	Cell line derived	KIT p.560-576 del exon 11	imatinib, sunitinib
GIST430/654	Cell line derived	KIT p.560-576 del exon 11<br>KIT p.V654A (exon 13)	sunitinib
GS11342	Patient derived	KIT p.WKV557fs (exon 11)	imatinib, sunitinib
GS11328	Patient derived	KIT p.WK557del (exon 11)	imatinib, sunitinib
GS11331	Patient derived	KIT p.WK557del (exon 11)<br>KIT p.V654A (exon 13)	sunitinib
GS5108	Patient derived	KIT p.WK557del (exon 11)<br>KIT p.Y823D homozygous (exon 17)	-
