# Supplementary material for: M4205 (IDRX-42) Is a Highly Selective and Potent Inhibitor of Relevant Oncogenic Driver and Resistance Variants of KIT in Cancer
Source: Mol Cancer Ther. 2025 Feb 28;24(7):1040–53. doi: 10.1158/1535-7163.MCT-24-0699 (PMC12214875; doi:10.1158/1535-7163.MCT-24-0699)
Supplement: Supplementary Table S2 — In vivo formulation and administration [file mct-24-0699_supplementary_table_s2_supps2.pdf]

Supplementary Table S2

Compound formulation and administration for in vivo studies.

	Vehicle formulation	Dosing
M4205	0.5% Methocel, 0.25% Tween20, 0.05M sodium citrate buffer, pH 3	once daily as indicated in graph
Imatinib	0.5% Methocel, 0.25% Tween20, 0.1M sodium citrate buffer, pH 4.5	50 mg/kg bi-daily
Sunitinib	0.5% Methocel, 0.25% Tween20, 0.1M sodium citrate buffer, pH 4.5	20 mg/kg 5d bi-daily, 2d daily
Ripretinib	0.5% Methocel, 0.25% Tween20, PBS, pH 7.4	75 mg/kg bi-daily
