# Supplementary material for: M4205 (IDRX-42) Is a Highly Selective and Potent Inhibitor of Relevant Oncogenic Driver and Resistance Variants of KIT in Cancer
Source: Mol Cancer Ther. 2025 Feb 28;24(7):1040–53. doi: 10.1158/1535-7163.MCT-24-0699 (PMC12214875; doi:10.1158/1535-7163.MCT-24-0699)
Supplement: Supplementary Table S3 — GENIE KIT mutation frequency [file mct-24-0699_supplementary_table_s3_supps3.pdf]

Supplementary Table S3

Mutation frequency in samples from GIST patients (AACR Project GENIE, Release 15.0-public, based on 1384 patients with full coverage of exons 9,11,13,14,17 and 18).

Protein Change	Exon	Count*	Frequency[#]
p.W557_K558del	11	111	12.1%
p.V559D	11	79	8.6%
p.V654A	13	65	65.0%
p.K642E	13	29	29.0%
p.N822K	17	33	23.6%
p.Y823D	17	24	17.1%
p.D820Y	17	21	15.0%

* Unique number of patients carrying the mutation. If multiple samples of one patient contain the mutation it is counted only once.
[#] Relative frequency of mutation in given exon that correspond to this protein change.
