# Supplementary material for: M4205 (IDRX-42) Is a Highly Selective and Potent Inhibitor of Relevant Oncogenic Driver and Resistance Variants of KIT in Cancer
Source: Mol Cancer Ther. 2025 Feb 28;24(7):1040–53. doi: 10.1158/1535-7163.MCT-24-0699 (PMC12214875; doi:10.1158/1535-7163.MCT-24-0699)
Supplement: Supplementary Table S4 — Biochemical inhibition of KIT variants [file mct-24-0699_supplementary_table_s4_supps4.pdf]

Supplementary Table S4

Inhibition of KIT mutant variants in vitro (average of two independent experiments, except for K642E variant).

	Biochemical pIC_{50}	M4205	Imatinib	Sunitinib	Regorafenib	Ripretinib	Avapritinib	NB003
KIT	wildtype	8.2	7.3	8.0	6.2	6.6	7.6	8.4
KIT (554-976) Exon 11 JM domain	del557-558	>9.3	8.2	7.9	7.2	9.0	9.3	9.2
	V559D	9.2	7.4	6.7	6.7	9.2	9.2	9.0
	V559A	>9.3	8.2	7.9	6.9	9.2	9.6	9.1
	V560G	8.9	7.1	6.6	6.6	8.5	9.0	8.9
KIT (554-976) Exon 13/14 ATP pocket	V559D/V654A	7.3	<5.0	7.3	5.1	<5	8.1	7.4
	V559D/T670I	<5.0	<5.0	7.0	5.9	6.3	7.5	6.2
	K642E	9	7.7	8	7.5	8.4	8.3	8.6
	V654A	7.5	5.7	8.0	6.2	6.7	6.6	7.5
	T670I	5.9	5.2	7.4	6.3	7	6.9	7.2
KIT (544-976) Exon 17/18 Activation loop	V560G/N822K	9.1	6.6	6.7	6.4	9.0	9.6	8.1
	V560G/D816V	7.1	5.1	6.9	5.7	9.0	9.8	5.7
	D816E	9.2	7.1	7.1	6.3	9.0	10.0	8.5
	D816F	7.3	4.9	6.9	6	9.0	9.7	5.9
	D816H	7.7	5.3	6.7	5.9	8.4	9.5	6.7
	D816V	6.5	5.1	6.8	5.4	8.5	10	5.4
	D816I	6.6	<5.0	6.7	5.3	9.1	11	5
	D816Y	7.8	5	6.8	5.8	8.7	9.8	6.4
	D820Y	9.2	7.3	7.9	6.7	8.9	9.3	8.8
	D820E	9	7.5	7.5	6.7	8.9	9.0	8.8
	Y823D	8.9	6.7	7.0	6.9	9.0	8.8	8.5
	A829P	9.2	7.1	6.8	6.6	9.0	9.4	8.8
