# Supplementary material for: M4205 (IDRX-42) Is a Highly Selective and Potent Inhibitor of Relevant Oncogenic Driver and Resistance Variants of KIT in Cancer
Source: Mol Cancer Ther. 2025 Feb 28;24(7):1040–53. doi: 10.1158/1535-7163.MCT-24-0699 (PMC12214875; doi:10.1158/1535-7163.MCT-24-0699)
Supplement: Supplementary Table S5 — Cellular P-KIT inhibition [file mct-24-0699_supplementary_table_s5_supps5.pdf]

Supplementary Table S5

Inhibition of KIT autophosphorylation (Y703) in cell lines.

	GIST430 (KIT exon 11 del.560-576) IC_{50} ± SD [nM]; n=2	GIST430/654 (KIT exon 11/13 del.560-576 / V654A) IC_{50} ± SD [nM]; n=2	Kasumi-1 (KIT exon 17 N822K) IC_{50} ± SD [nM]; n=2
M4205	4 ±1	48 ± 21	4 ±1
Imatinib	59 ± 5	4750 ± 1750	784 ± 17
Sunitinib	27 ± 8	70 ± 31	440 ± 70
Regorafenib	131 ± 83	1500 ± 707	192 ± 11
Ripretinib	15 ± 1	185 ± 5.5	51 ± 2
Avapritinib	54 ± 4	1650 ± 778	190 ± 28
NB003	3 ± 0	43 ± 30	8 ± 4
