# Supplementary material for: M4205 (IDRX-42) Is a Highly Selective and Potent Inhibitor of Relevant Oncogenic Driver and Resistance Variants of KIT in Cancer
Source: Mol Cancer Ther. 2025 Feb 28;24(7):1040–53. doi: 10.1158/1535-7163.MCT-24-0699 (PMC12214875; doi:10.1158/1535-7163.MCT-24-0699)
Supplement: Supplementary Table S6 — Cellular viability inhibition [file mct-24-0699_supplementary_table_s6_supps6.pdf]

Supplementary Table S6

Inhibition of viability in cell lines.

	GIST430 (KIT exon 11 del.560-576) IC_{50} ± SD [nM]; n=2	GIST430/654 (KIT exon 11/13 del.560-576 / V654A) IC_{50} ± SD [nM]; n=2	Kasumi-1 (KIT exon 17 N822K) IC_{50} ± SD [nM]; n=2	GIST48B (KIT wildtype) IC_{50} ± SD [nM]; n=2
M4205	2 ± 0.5	21 ± 6	2 ± 0.1	16250 ± 10750
Imatinib	18 ± 4	1032 ± 168	205 ± 5	> 30000
Sunitinib	15 ± 1	41 ± 0.5	13 ± 0	10600 ± 2525
Ripretinib	5 ± 0.4	38 ± 4	5 ± 0.4	715 ± 71
Avapritinib	48 ± 4	121 ± 1	13 ± 0	8400 ± 1556
NB003	1 ± 0	10 ± 2	2 ± 0	14000 ± 2828
