# Supplementary material for: M4205 (IDRX-42) Is a Highly Selective and Potent Inhibitor of Relevant Oncogenic Driver and Resistance Variants of KIT in Cancer
Source: Mol Cancer Ther. 2025 Feb 28;24(7):1040–53. doi: 10.1158/1535-7163.MCT-24-0699 (PMC12214875; doi:10.1158/1535-7163.MCT-24-0699)
Supplement: Supplementary Table S7 — Mouse Histopathology [file mct-24-0699_supplementary_table_s7_supps7.pdf]

Supplementary Table S7

Histopathological assessment: heart, liver, lung, spleen, thymus, kidney, bone (knee joint), bone marrow (sternum + femur), adrenal, brain, eye, optic nerve, gastro-intestinal tract, and female reproductive tract (ovary, uterus, vagina) were fixed in 4% formalin and processed for histopathology. Hematoxylin- and Eosin-stained sections were examined by light microscopy. Only organs where findings were observed are listed. N=3-4 mice per group.

**Finding**	**Vehicle 31d**	**17.5 mg/kg**	**35 mg/kg 31d**	**75 mg/kg 22d**	**150 mg/kg 7d**
Spleen: extramedullary hematopoiesis	none	none	Mild in 2/3	Mild in 1/3, moderate in 2/3	Moderate in all
Bone marrow: hematopoietic celullarity decrease	none	none	Minimal in 2/3	Moderate in 3/3	Moderate in 4/4
Lung: foamy alveolar macrophages	none	none	none	None	Minimal in all
Kidney: hypertrophy	none	none	none	Mild in 2/3 animals	Moderate in 3/4 animals
GI Tract: ulceration	none	none	none	none	Marked in 2/4
