# Supplementary material for: M4205 (IDRX-42) Is a Highly Selective and Potent Inhibitor of Relevant Oncogenic Driver and Resistance Variants of KIT in Cancer
Source: Mol Cancer Ther. 2025 Feb 28;24(7):1040–53. doi: 10.1158/1535-7163.MCT-24-0699 (PMC12214875; doi:10.1158/1535-7163.MCT-24-0699)
Supplement: Supplementary Table S8 — Mouse Hematology [file mct-24-0699_supplementary_table_s8_supps8.pdf]

Supplementary Table S8 Hematology in mice

Hematology was assessed in mouse blood after addition of EDTA as anti-coagulant, using an ADVIA 120 Autoanalyzer. The following parameters were assessed: RBC: Red Blood Cell count, HGB: Hemoglobin concentration, HCT: Hematocrit, MCV: Mean Corpuscular Volume, MCHC: Mean Corpuscular Hemoglobin Concentration, PLT: Platelet count, RET: Reticulocyte count, WBC: White Blood Cell count, NEU: Neutrophil count, LYM: Lymphocyte count, EOS: Eosinophil count, BASO: Basophil count, MONO: Monocyte count, LUC: Large Unstained Cells count. LSV: Low Sample Volume.

Values labeled in red indicate deviation from normal range.

Group	Value	RBC [/pL]	HGB [g/dL]	HCT [%]	MCV [fL]	MCH [pg]	MCHC [g/dL]	PLT [/nL]	RET [%]	WBC [/nL]	NEU [%]	LYM [%]	EOS* [%]	BASO* [%]	MONO* [%]	LUC* [%]
Vehicle Control	Animal 1	10,36	16,0	49,2	47,5	15,4	32,5	1030	2,57	0,76	43,5	44,4	6,3	0,3	6,3	0,6
	Animal 2	10,14	15,8	48,4	47,8	15,5	32,4	962	2,23	0,58	68,9	20,4	4,1	0,8	5,8	0,0
	Animal 3	9,80	15,0	48,0	49,0	15,4	31,4	900	3,30	0,70	66,5	21,4	6,0	0,0	6,0	0,0
	Animal 4	9,86	15,2	48,0	48,7	15,4	31,6	852	3,03	0,36	47,6	30,8	7,0	0,0	12,6	2,1
	Animal 5	10,22	15,6	49,0	48,0	15,2	31,7	1004	3,55	0,66	56,4	30,0	3,6	0,0	9,9	0,0
	**Mean**	**10,08**	**15,5**	**48,5**	**48,2**	**15,4**	**31,9**	**950**	**2,94**	**0,61**	**56,6**	**29,4**	**5,4**	**0,2**	**8,1**	**0,5**
	SD	0,214	0,37	0,50	0,56	0,10	0,44	65,7	0,480	0,139	10,02	8,63	1,32	0,31	2,70	0,81
	Median	10,14	15,6	48,4	48,0	15,4	31,7	962	3,03	0,66	56,4	30,0	6,0	0,0	6,3	0,0
35 mg/kg QD	Animal 1	clot	clot	clot	clot	clot	clot	clot	clot	clot	clot	clot	clot	clot	clot	clot
	Animal 2	clot	clot	clot	clot	clot	clot	clot	clot	clot	clot	clot	clot	clot	clot	clot
	Animal 3	LSV	LSV	LSV	LSV	LSV	LSV	LSV	LSV	LSV	LSV	LSV	LSV	LSV	LSV	LSV
	Animal 4	6,60	11,4	36,8	55,8	17,4	31,1	1742	3,13	0,28	57,1	19,2	17,5	0,9	4,5	0,0
	Animal 5	6,58	11,4	36,4	55,4	17,3	31,2	1664	2,81	0,22	38,1	8,2	47,8	0,0	4,5	1,5
	**Mean**	**6,59**	**11,4**	**36,60**	**55,6**	**17,35**	**31,2**	**1703**	**2,97**	**0,3**	**47,60**	**13,7**	**32,65**	**0,5**	**4,50**	**0,8**
	SD	0,010	0,00	0,200	0,20	0,050	0,05	39,00	0,160	0,03	9,500	5,50	15,150	0,45	0,000	0,75
	Median	6,59	11,4	36,60	55,6	17,35	31,2	1703,0	3,0	0,3	47,60	13,7	32,65	0,5	4,50	0,8
75 mg/kg QD	Animal 1	4,08	6,2	20,0	49,1	15,2	31,0	648	1,34	0,18	57,7	15,5	20,6	0,0	5,2	1,0
	Animal 2	6,28	10,0	31,2	49,8	15,9	32,0	1106	0,90	0,28	50,5	30,2	16,5	2,5	1,1	1,6
	Animal 3	5,48	9,2	28,8	52,4	17,0	32,2	1012	2,41	0,10	59,5	17,6	18,9	1,9	2,7	1,4
	Animal 4	6,26	9,8	30,8	49,1	15,7	31,9	1104	1,20	0,20	58,5	17,1	17,1	0,0	4,9	2,4
	Animal 5	6,62	11,0	34,2	51,5	16,7	32,3	1054	1,59	0,20	61,7	11,1	21,0	1,2	6,2	0,0
	**Mean**	**5,74**	**9,2**	**29,0**	**50,4**	**16,1**	**31,9**	**985**	**1,49**	**0,2**	**57,6**	**18,3**	**18,8**	**1,1**	**4,0**	**1,3**
	SD	0,912	1,63	4,82	1,34	0,66	0,46	172,0	0,512	0,057	3,79	6,38	1,80	1,00	1,85	0,79
	Median	6,26	9,8	30,8	49,8	15,9	32,0	1054	1,34	0,2	58,5	17,1	18,9	1,2	4,9	1,4
