# Supplementary material for: M4205 (IDRX-42) Is a Highly Selective and Potent Inhibitor of Relevant Oncogenic Driver and Resistance Variants of KIT in Cancer
Source: Mol Cancer Ther. 2025 Feb 28;24(7):1040–53. doi: 10.1158/1535-7163.MCT-24-0699 (PMC12214875; doi:10.1158/1535-7163.MCT-24-0699)
Supplement: Supplementary Table S9 — Mouse Clinical Chemistry [file mct-24-0699_supplementary_table_s9_supps9.pdf]

Supplementary Table S9 Clinical Chemistry in mice

Clinical chemistry assessment of Calcium, Inorganic Phosphate, Glucose, Urea, Creatinine, Total Bilirubin, Cholesterol, Triglycerides, Total Protein, Albumin, Alanine Aminotransferase, Aspartate Aminotransferase, Alkaline Phosphatase was assessed in serum of mice harvested from full blood after coagulation. An ADVIA 1800 Autoanalyzer was used. Values labeled in red indicate deviation from normal range.

Group	animal	time after treatment [h]	Ca mmol/L	IP mmol/L	GLUC mmol/L	UREA mmol/L	CREA µmol/L	TBIL µmol/L	CHOL mmol/L	TRIG mmol/L	TP G/L	ALB G/L	ALB/ Glob-Ratio	ALAT U/L	ASAT U/L	AP U/L
**Vehicle**	1	2	2,29	2,45	8,9	7,0	3	0,6	2,78	0,85	47,8	33,4	2,32	29	68	67
	2	2	2,34	3,62	11,2	8,2	8	1,1	2,63	0,57	44,9	31,7	2,40	34	66	60
	3	2	2,30	3,57	13,7	6,9	6	0,3	2,34	0,99	44,4	30,8	2,26	30	62	62
	4	5	2,33	4,30	13,0	9,0	12	1,2	2,61	0,63	43,3	30,4	2,36	33	67	50
	5	5	2,17	3,39	17,2	8,9	7	0,1	2,49	0,97	42,5	29,9	2,37	81	83	56
Mean			**2,29**	**3,47**	**12,8**	**8,0**	**7**	**0,7**	**2,57**	**0,80**	**44,6**	**31,2**	**2,34**	**41**	**69**	**59**
SD			0,061	0,595	2,76	0,90	2,9	0,43	0,147	0,173	1,81	1,23	0,047	19,9	7,2	5,7
Median			2,30	3,57	13,0	8,2	7	0,6	2,61	0,85	44,4	30,8	2,36	33	67	60
**35 mg/kg QD**	1	2	2,25	2,74	13,2	9,2	7	0,5	2,24	1,04	44,8	32,3	2,58	59	116	74
	2	2	2,16	1,42	10,4	8,1	8	1,7	2,30	0,71	45,5	31,9	2,35	47	123	75
	3	2	2,31	2,89	14,5	8,0	11	0,4	2,35	1,39	42,7	30,2	2,42	37	213	68
	4	5	2,16	2,97	17,6	6,9	3	0,7	1,88	0,63	37,8	27,0	2,50	38	89	63
	5	5	2,13	3,11	19,1	7,6	1	1,1	2,30	1,03	42,5	30,4	2,51	31	65	61
Mean			**2,20**	**2,63**	**15,0**	**8,0**	**6**	**0,9**	**2,21**	**0,96**	**42,7**	**30,4**	**2,47**	**42**	**121**	**68**
SD			0,067	0,615	3,11	0,75	3,6	0,47	0,171	0,271	2,69	1,87	0,083	9,7	50,3	5,6
Median			2,16	2,89	14,5	8,0	7	0,7	2,30	1,03	42,7	30,4	2,50	38	116	68
**75 mg/kg QD**	1	2	2,12	2,74	18,2	5,9	6	1,1	2,98	0,62	40,8	27,5	2,07	27	50	68
	2	2	2,34	5,08	16,2	5,9	9	1,3	3,72	0,65	45,2	31,6	2,32	69	113	110
	3	2	2,08	3,52	21,3	6,2	12	1,7	3,03	1,08	41,9	28,2	2,06	27	57	94
	4	5	2,11	2,00	10,6	5,6	4	1,7	2,68	0,71	40,2	27,5	2,17	51	111	94
	5	5	2,16	2,39	12,2	6,9	9	1,2	3,13	0,68	41,9	28,4	2,10	27	71	93
Mean			**2,16**	**3,15**	**15,7**	**6,1**	**8**	**1,4**	**3,11**	**0,75**	**42,0**	**28,6**	**2,14**	**40**	**80**	**92**
SD			0,093	1,089	3,90	0,44	2,8	0,25	0,341	0,169	1,73	1,52	0,097	17,1	26,7	13,5
Median			2,12	2,74	16,2	5,9	9	1,3	3,03	0,68	41,9	28,2	2,10	27	71	94
