# Supplementary material for: M4205 (IDRX-42) Is a Highly Selective and Potent Inhibitor of Relevant Oncogenic Driver and Resistance Variants of KIT in Cancer
Source: Mol Cancer Ther. 2025 Feb 28;24(7):1040–53. doi: 10.1158/1535-7163.MCT-24-0699 (PMC12214875; doi:10.1158/1535-7163.MCT-24-0699)
Supplement: Supplementary Table S10 — Split of M4205 concentration and FLT3 inhibition [file mct-24-0699_supplementary_table_s10_supps10.pdf]

Supplementary Table S10

Split of predicted human free C_{avg} of M4205 and FLT3 inhibition.

FLT3 assay	M4205 IC_{50} [nM]	Predicted c_{avg} of M4205 at 260 mg QD [nM]	Split FLT3 IC_{50} / C_{avg}
Biochemical inhibition	262	16	16
NanoBRET target engagement	263		16
Mv-4-11 viability	81		5
MOLM-13 viability	101		6
